# Supplementary material for: POINT technology illuminates the processing of polymerase-associated intact nascent transcripts
Source: Mol Cell. 2021 May 6;81(9):1935–1950.e6. doi: 10.1016/j.molcel.2021.02.034 (PMC8122139; doi:10.1016/j.molcel.2021.02.034)
Supplement: Document S1. Figures S1–S7 [file mmc1.pdf]

Molecular Cell, Volume *81*

Supplemental information

POINT technology illuminates the processing of polymerase-associated intact nascent transcripts

Rui Sousa-Luís, Gwendal Dujardin, Inna Zukher, Hiroshi Kimura, Carika Weldon, Maria Carmo-Fonseca, Nick J. Proudfoot, and Takayuki Nojima

SUPPLEMENTARY INFORMATION

POINT TECHNOLOGY ILLUMINATES THE PROCESSING OF POLYMERASE-ASSOCIATED INTACT NASCENT TRANSCRIPTS

Rui Sousa- Luís[1, 6], Gwendal Dujardin[2], Inna Zukher[2], Hiroshi Kimura[3], Carika Weldon[4], Maria Carmo-Fonseca[1,7], Nick J Proudfoot[2,7], and Takayuki Nojima[2,5,6,7,8]

[1]Instituto de Medicina Molecular João Lobo Antunes, Faculdade de Medicina, Universidade de Lisboa, Av. Professor Egas Moniz 1649-028, Lisboa, Portugal
[2]Sir William Dunn School of Pathology, University of Oxford, South Parks Road, Oxford, OX1 3RE, UK
[3]Cell Biology Centre, Tokyo Institute of Technology, 4259 Nagatsuta-cho, Midori-ku, Yokohama, Kanagawa 226-8503, Japan
[4]Wellcome Trust Center for Human Genetics, University of Oxford, Roosevelt Drive, OX3 7BN, UK
[5]Medical institute of Bioregulation, Kyushu University, 3-1-1 Maidashi, Higashi-ku, Fukuoka, 812-8582, Japan
[6]Co-first authors
[7]Co-corresponding authors
[8]Lead contact

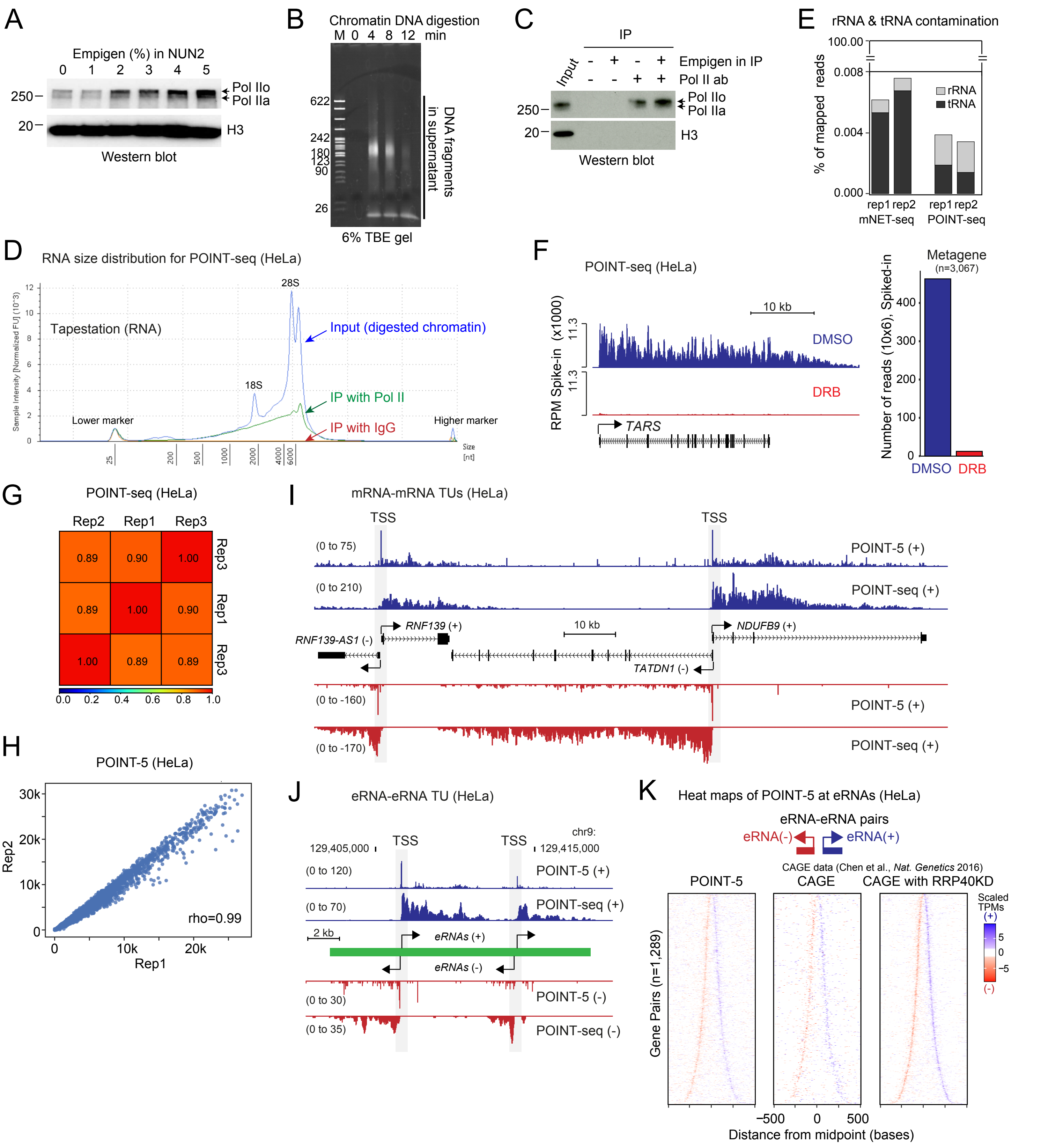

Figure S1. Additional data for POINT-seq and POINT-5 methods (Related to Figure 1)
(A) Western blot of supernatant from DNase-digested HeLa chromatin treated with indicated concentration of Empigen in NUN2 buffer. Antibodies used against Pol II CTD (CMA601) and histone H3. Pol IIo denotes phosphorylated while Pol IIa denotes unphosphorylated isoforms
(B) Gel image of DNA fragments in supernatant of DNase-digested HeLa chromatin treated with 3% Empigen in NUN2 for indicated incubation times.
(C) Western blot of supernatant from DNase-digested HeLa chromatin (input) and IP with anti-Pol II CTD antibody. Antibodies for western blot were anti-Pol II CTD (CMA601) and anti-histone H3.
(D) Tapestation image of POINT RNA size distribution. Digested chromatin fraction as input (blue), IP with mouse IgG (red), and IP with anti-Pol II CTD antibody CMA601 (green).
(E) Stacked barplot showing the percentage of mapped reads overlapping rRNA and tRNA TUs for both mNET-seq and POINT-seq.
(F) (Left) *TARS* of example of POINT-seq signals and (Right) quantification of POINT-seq metagene signals of nonoverlapping PC genes (n=3067) from DMSO or DRB (4 h) -treated HeLa cells.
(G) Heat map for reproducibility between three biological replicates of POINT-seq in HeLa cells.
(H) Scatter plot for reproducibility between two biological replicates of POINT-5 in HeLa cells.
(I) Example view of POINT-5 and POINT-seq signals for mRNA-mRNA pairs in HeLa cells.
(J) Example view of POINT-5 and POINT-seq signals for eRNA-eRNA pairs in HeLa cells.
(K) Heat maps of POINT-5 and CAGE (-/+ RRP40KD) for eRNA-eRNA pairs in HeLa cells. Scaled Transcripts Per Million (TPM) is shown for sense (+, blue) and antisense (-, red).

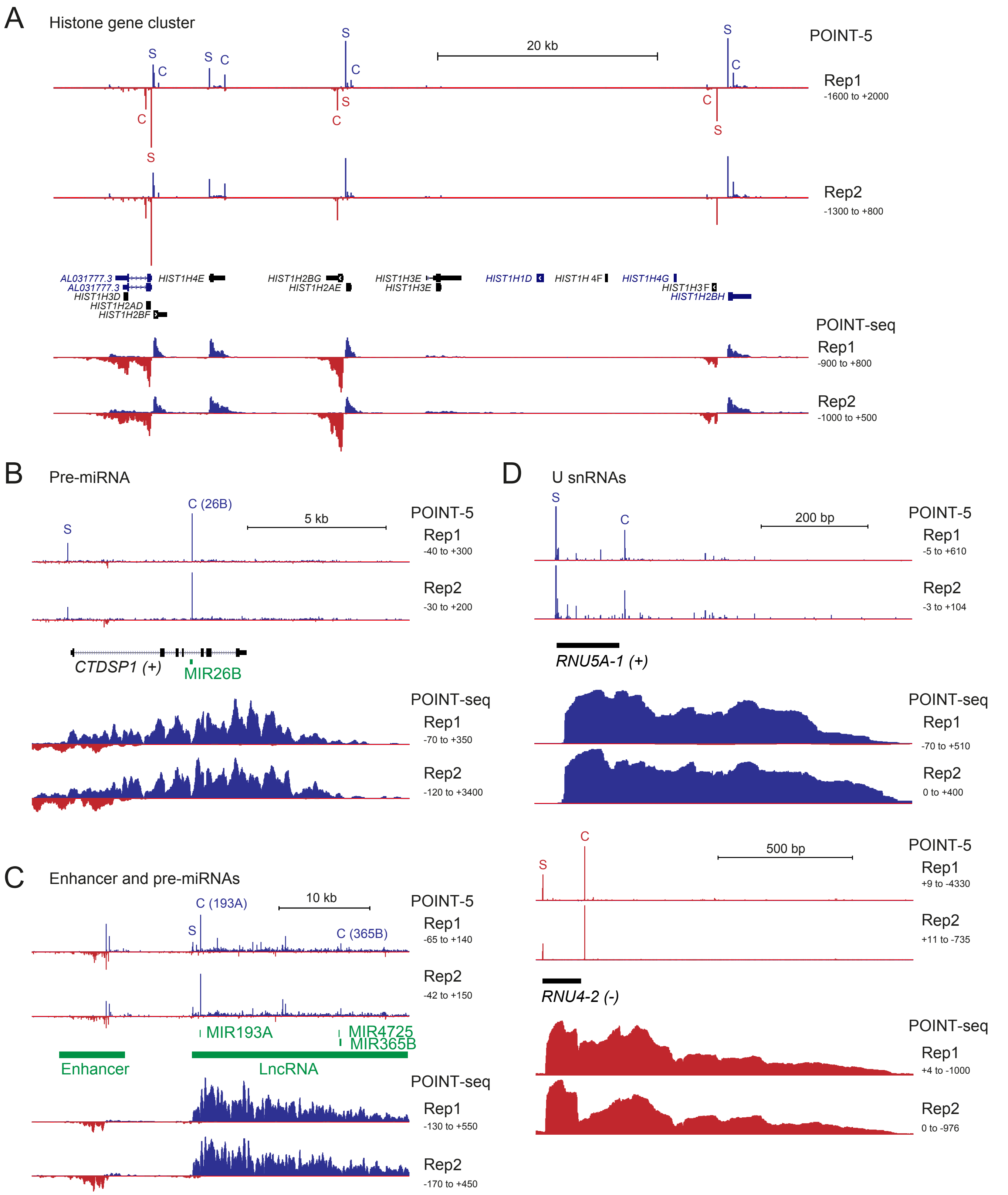

Figure S2. Duplicates of POINT-5 and POINT-seq gene profiles (Related to Figure 2)
(A-D) Example views of POINT-5 and POINT-seq signals on (A) Histone gene cluster, (B) *CTDSP1* as miR26B host gene, (C) Enhancer and unannotated lncRNA with pre-miRNAs, and (D) *RNU5A-1* and *RNU4-2* as U snRNA genes.
Sense strand, blue. Antisense strand, red. S and C are represented as transcription start and RNA cleavage sites, respectively.

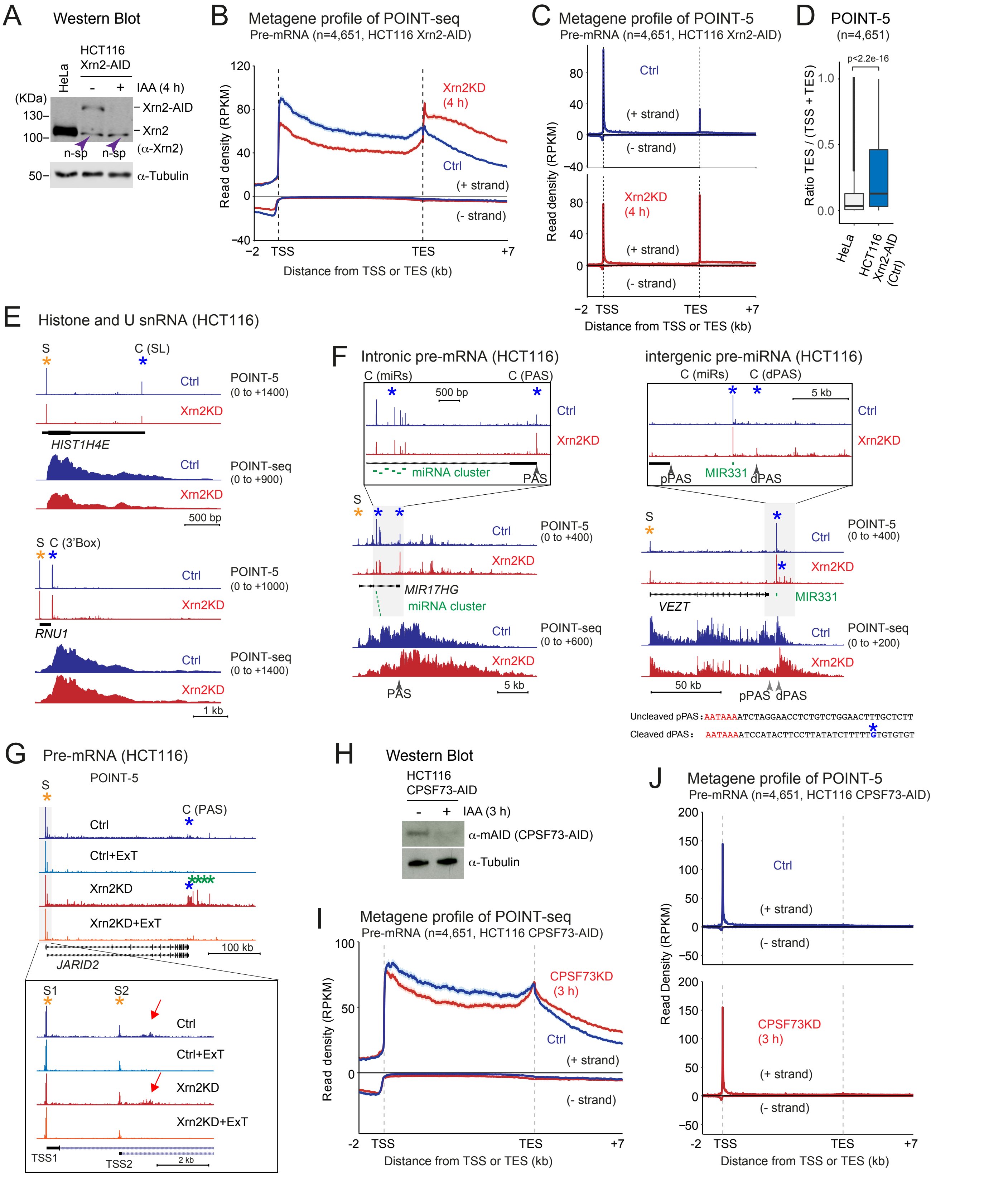

Figure S3. Metagene profile and example genes of POINT-seq and POINT-5 in Xrn2 and CPSF-73 depleted HCT116 cells (Related to Figure 3)

(A) Western blot of whole cell extract from HeLa and HCT116 Xrn2-AID (-/+ IAA, 4 h). Antibodies against Xrn2 and Tubulin. Non-specific (n-sp) bands from anti-Xrn2 antibody indicated by arrow heads.

(B and C) Metagene profile of POINT-seq (B) or POINT-5 (C) on normalised PC genes in HCT116 Xrn2-AID. Ctrl (control) denotes -IAA, KD denotes +IAA for 4 h.

(D) Ratio of TES and TSS on PC genes in HeLa and HCT116 Xrn2-AID Ctrl cells.

(E) *HIST1H4E* and *RNU1* as examples of POINT-5 and POINT-seq on histone and U snRNA genes in HCT116 Xrn2-AID Ctrl and KD cells.

(F) *MIR17HG* and *VEZT* as examples of POINT-5 and POINT-seq on miRNA host genes in HCT116 Xrn2-AID Ctrl and KD cells.

(G) *JARID2* as example of POINT-5 and POINT-seq on PC gene in HCT116 Xrn2-AID Ctrl and KD cells and in vitro ExT treatment. POINT-5 peaks located downstream of PAS in HCT116 Xrn2-AID (KD) cells are indicated as green asterisks. ExT-sensitive POINT-5 peaks located downstream of TSS2 indicated by red arrows.

(H) Western blot of whole cell extract of HeLa and HCT116 CPSF73-AID (-/+ IAA, 3 h). Antibodies against mAID and Tubulin.

(I and J) Metagene profile of (I) POINT-seq or (J) POINT-5 on normalised PC genes (n=4,651) in HCT116 CPSF73-AID Ctrl and KD cells.

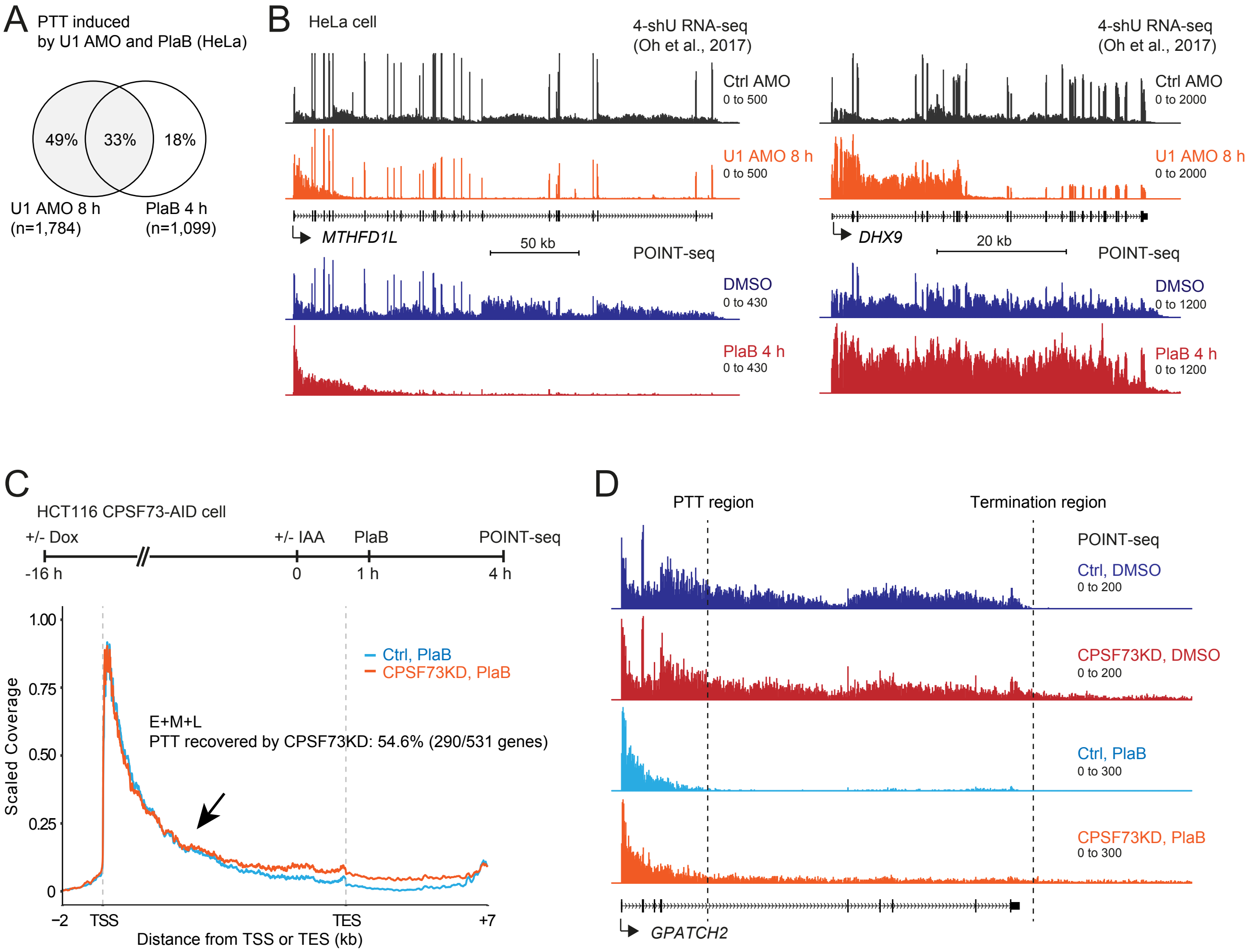

Figure S4. Additional analysis of PlaB induced PTT (Related to Figure 4)
(A) Venn graph of PTT in genes induced by U1 AMO (8 h) or PlaB (4 h) treatment in HeLa cells.
(B) *MTHFD1L* and *DHX9* as examples of 4-shU RNA-seq (Oh et al., 2017) and POINT-seq (this study) signals.
(C) (Top) Schematic of CPSF73KD and PlaB treatment in HCT116 CPSF73-AID cells. (Bottom) Metagene profile of POINT-seq with PlaB (light blue) or CPSF73KD and PlaB (orange) in normalized region from TSS-2kb to TES+7kb. Arrow indicates a starting point of PTT defect induced by CPSF73KD. 290 out of 531 PTT genes were affected.
(D) *GPATCH2* as example of POINT-seq for indicated treatments. The PTT and termination regions are shown as dashed lines.

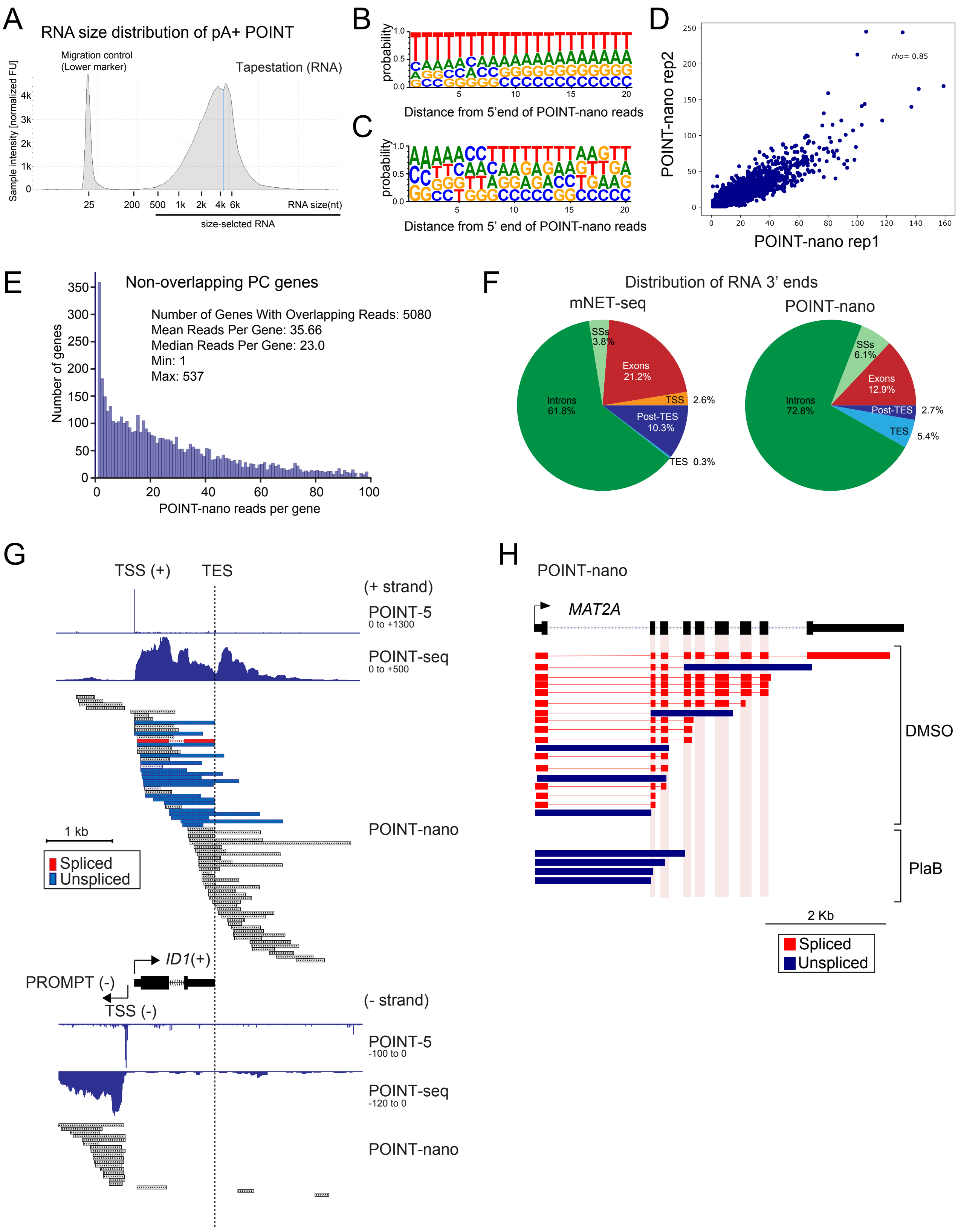

Figure S5. Additional characterization of POINT-nano (Related to Figure 5)
(A) Tapestation image of size distribution of in vitro polyadenylated POINT RNA. The RNA (>500 nt) were size-selected before ONT library prep.
(B and C) Consensus sequences of POINT-nano 5' end reads (B) before or (C) after removal of internal polyA tracts (reverse complement).
(D) Scatter plot for reproducibility between two biological replicates of POINT-nano signals in HeLa cells.
(E) PC gene coverage of POINT-nano reads.
(F) Pie charts of mNET-seq and POINT-nano RNA 3' ends for positions of PC genes.
(G) *ID1* as example of POINT-5, POINT-seq, and POINT-nano signals. Spliced (red), unspliced (blue), other (grey) reads.
(H) POINT-nano reads for *MAT2A* as example of a short PC gene displaying high levels of immediate splicing. Spliced (red), Unspliced (blue).

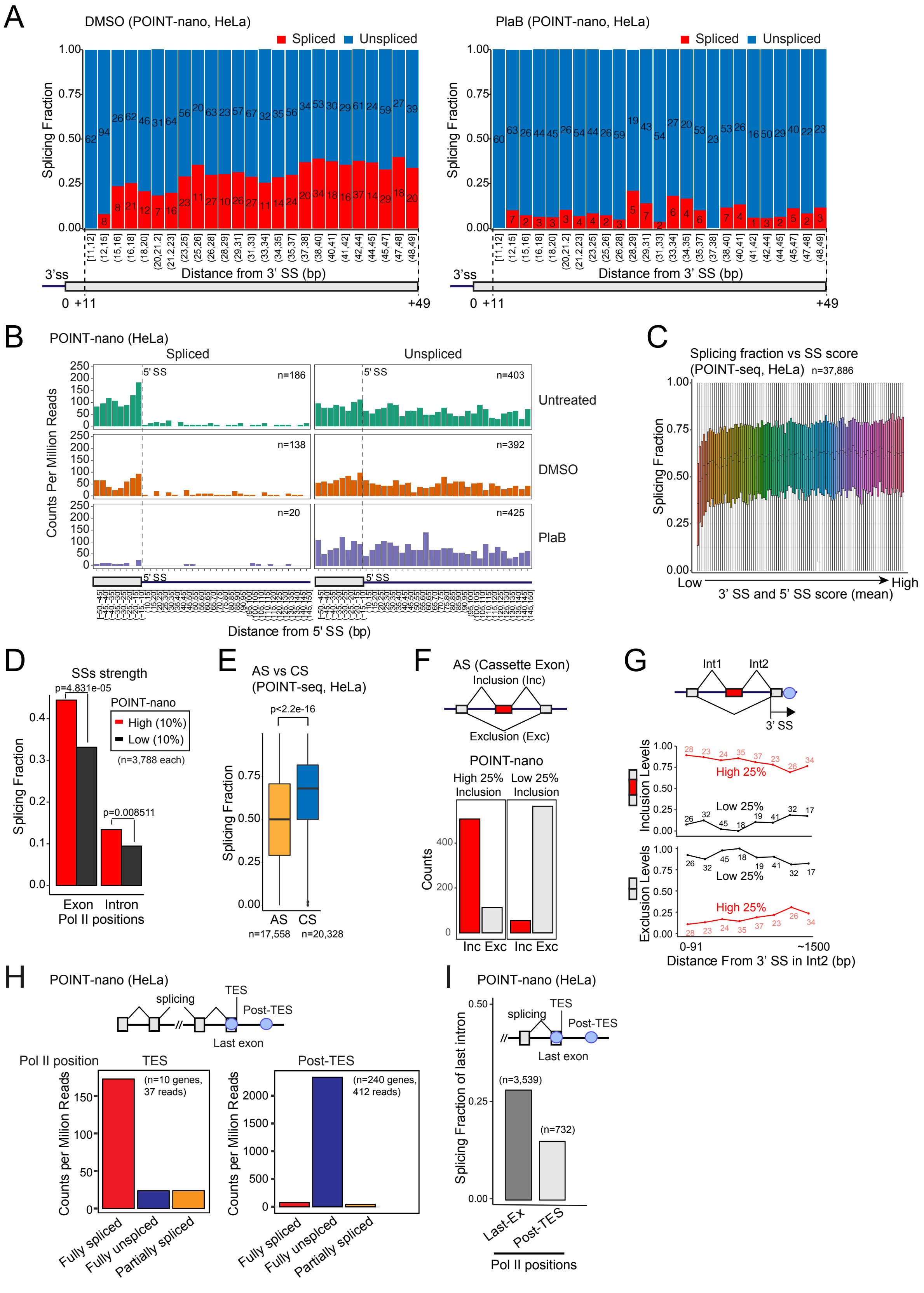

Figure S6. Additional bioinformatics on co-transcriptional splicing kinetics (Related to Figure 6)
(A) Splicing fraction of POINT-nano reads with Pol II located at 3' SS+10 to +49 bp in HeLa cells. Spliced (red), Unspliced (blue).
(B) Counts per million of spliced and unspliced POINT-nano reads when Pol II is located at 5' SS-50 bp to 5' SS+150 bp are shown.
POINT-nano reads from Untreated (Unt, green), DMSO (orange) and PlaB (purple) treated HeLa cells were compared. 5' SSs are indicated by dashed lines. Bin size, 5 bp.
(C) Effect of 3' SS and 5' SS scores (mean) on splicing fraction in POINT-seq analyses.
(D) Effect of 3' SS and 5' SS scores (high 10% or low 10%) on splicing fraction in POINT-nano analyses with Pol II in exon and intron.
(E) Splicing fraction of POINT-seq signals for alternative splicing (AS) or constitutive splicing (CS).
(F) (Top) Cassette exon splicing events classified based on exon inclusion percentage using pA+ RNA-seq data.
(Bottom) Number of exon inclusion (red) and exclusion (grey) events in POINT-nano analyses profiled for high or low 25% exon inclusion event categories.
(G) Exon inclusion or exclusion levels in POINT-nano analyses with Pol II located downstream of 3' SS in intron 2 (Int2). High 25% and low 25% of exon inclusion events are shown as red and black lines, respectively. Numbers of splicing events are indicated.
(H) Counts per million of fully spliced (red), fully unspliced (blue) and partially spliced (orange) POINT-nano reads when Pol II is located at TES or Post-TES region of short genes (<1,500bp) are shown. 10 genes (37 reads) for TES and 240 genes (412 reads) were analysed.
(I) Splicing fraction for last intron removal in POINT-nano analyses. Pol II positions indicated below.

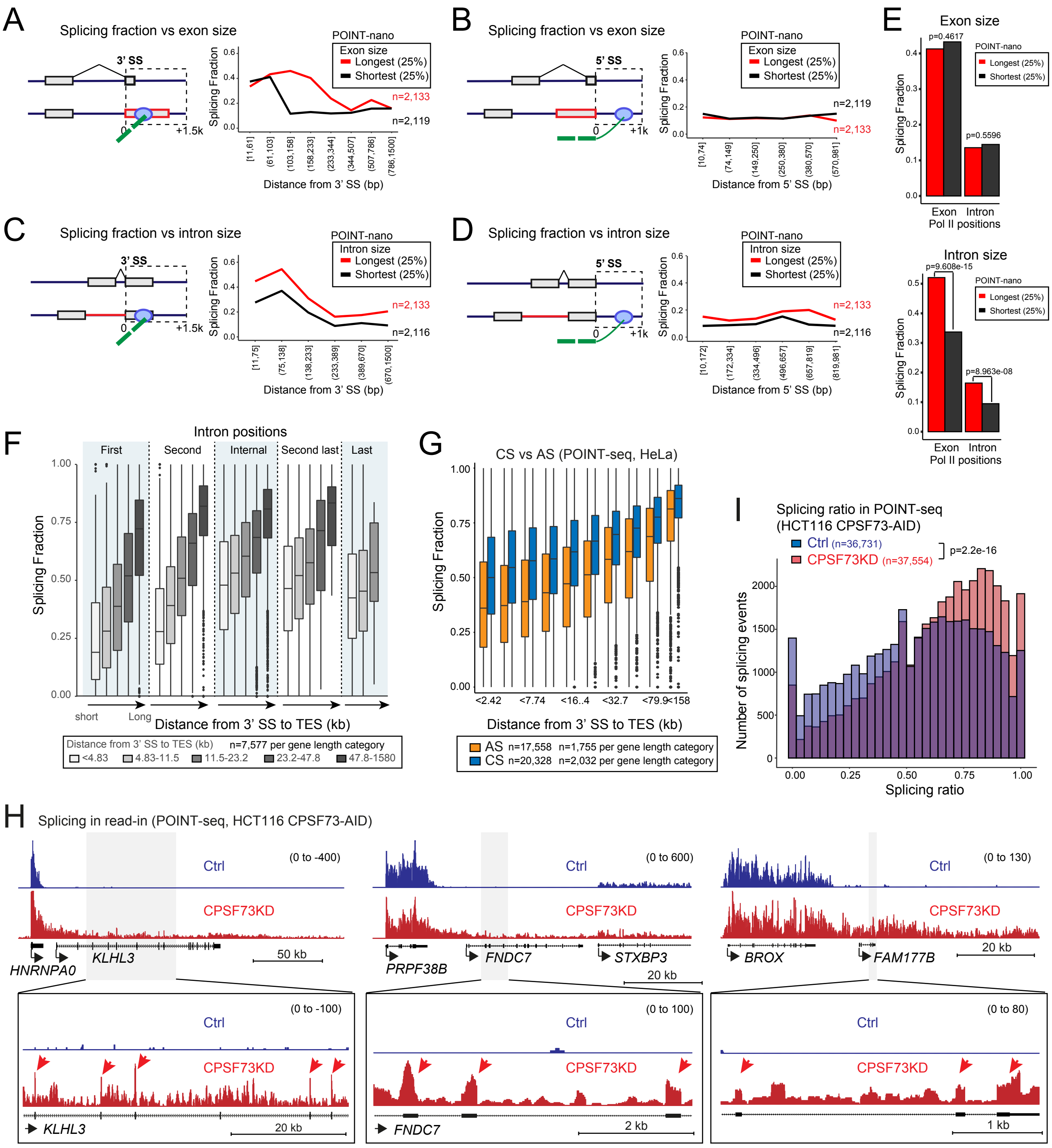

Figure S7. Additional bioinformatics on TU length effect on co-transcriptional splicing (Related to Figure 7)
(A-D) Effect of (A and B) exon or (C and D) intron sizes (long 25%, red or short 25%, black) on splicing fraction in POINT-nano analyses. Pol II is located downstream of (A and C) 3' SS (5' SS+0~1.5 kb) or (B and D) 5' SS (5' SS+0~1 kb).
(E) Quantification of effect of exon (top) or intron (bottom) sizes on splicing fraction POINT-nano signals. Pol II positions are indicated at bottom.
(F) Splicing fraction of POINT-seq signals in HeLa cells. Intron positions and distance from 3' SS to TES (kb) are indicated at top and bottom, respectively.
(G) Splicing fraction of POINT-seq signals in HeLa cells. Distance from 3' SS to TES (kb) is indicated below. AS (yellow), CS (blue).
(H) Examples of read-in transcripts shown by POINT-seq in HCT116 CPSF73-AID cells (-/+ IAA 3 h). Control (blue), CPSF73KD (red). CPSF73KD induced read-in of normally silent *KLHL3*, *FNDC7* and *FAM177B* by active upstream tandem PC genes. Exonic signals derived from splicing are indicated by red arrows.
(I) Number of introns with splicing ratio of POINT-seq signals in HCT116 CPSF73-AID cells (-/+ IAA 3 h). Control (blue), CPSF73KD (light red).
