# Supplementary material for: POINT technology illuminates the processing of polymerase-associated intact nascent transcripts
Source: Mol Cell. 2021 May 6;81(9):1935–1950.e6. doi: 10.1016/j.molcel.2021.02.034 (PMC8122139; doi:10.1016/j.molcel.2021.02.034)
Supplement: Document S2. Article plus supplemental information [file mmc2.pdf]

Molecular Cell

Article

POINT technology illuminates the processing of polymerase-associated intact nascent transcripts

Graphical abstract

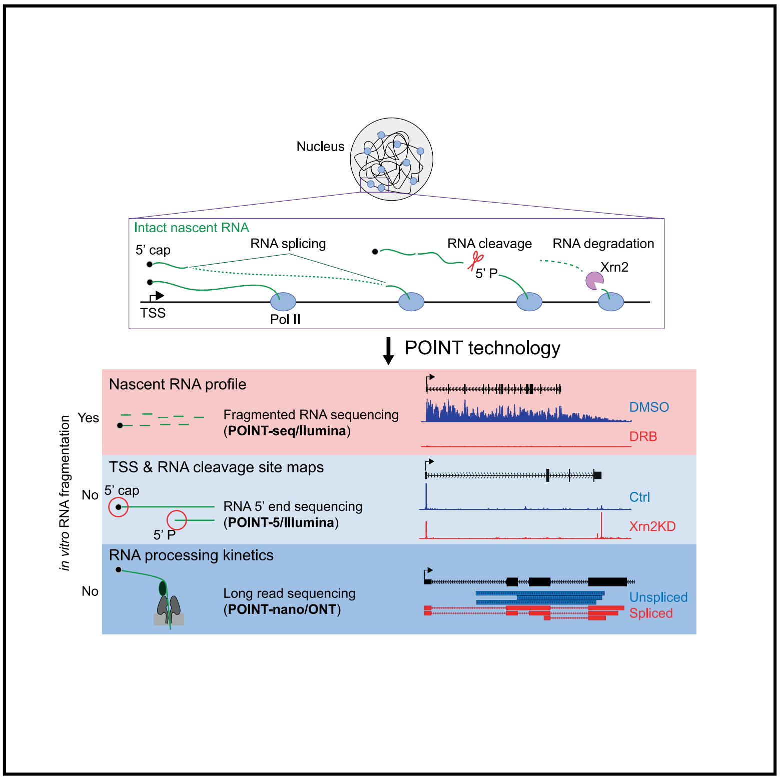

Highlights

- POINT methodology dissects intact nascent RNA processing
- Specificity of Xrn2 exonuclease in co-transcriptional RNA degradation
- Splicing suppresses Xrn2-dependent premature termination
- Different kinetic classes of co-transcriptional splicing in human genes

Authors

Rui Sousa-Luís, Gwendal Dujardin, Inna Zukher, ..., Maria Carmo-Fonseca, Nick J. Proudfoot, Takayuki Nojima

Correspondence

carmo.fonseca@medicina.ulisboa.pt (M.C.-F.),
nicholas.proudfoot@path.ox.ac.uk (N.J.P.),
taka.nojima@path.ox.ac.uk (T.N.)

In brief

Sousa-Luís et al. describe tripartite methodology to purify and sequence RNA polymerase II-associated intact nascent transcripts (POINT) from mammalian genomes. POINT-5 distinguishes nascent RNA 5′ end caps from 5′ end cleavage, POINT-seq profiles full-length transcription units, and POINT-nano determines the kinetics of co-transcriptional splicing at a single-molecule level.

Sousa-Luís et al., 2021, Molecular Cell *81*, 1935–1950
May 6, 2021 © 2021 The Author(s). Published by Elsevier Inc.
https://doi.org/10.1016/j.molcel.2021.02.034

Molecular Cell

CellPress
OPEN ACCESS

Article

POINT technology illuminates the processing of polymerase-associated intact nascent transcripts

Rui Sousa-Luís,[1,6] Gwendal Dujardin,[2] Inna Zukher,[2] Hiroshi Kimura,[3] Carika Weldon,[4] Maria Carmo-Fonseca,[1,*] Nick J. Proudfoot,[2,*] and Takayuki Nojima[2,5,6,7,*]
[1]Instituto de Medicina Molecular João Lobo Antunes, Faculdade de Medicina, Universidade de Lisboa, Av. Professor Egas Moniz, 1649-028 Lisbon, Portugal
[2]Sir William Dunn School of Pathology, University of Oxford, South Parks Road, Oxford OX1 3RE, UK
[3]Cell Biology Centre, Tokyo Institute of Technology, 4259 Nagatsuta-cho, Midori-ku, Yokohama, Kanagawa 226-8503, Japan
[4]Wellcome Trust Center for Human Genetics, University of Oxford, Roosevelt Drive, Oxford OX3 7BN, UK
[5]Medical Institute of Bioregulation, Kyushu University, 3-1-1 Maidashi, Higashi-ku, Fukuoka 812-8582, Japan
[6]These authors contributed equally
[7]Lead contact
*Correspondence: carmo.fonseca@medicina.ulisboa.pt (M.C.-F.), nicholas.proudfoot@path.ox.ac.uk (N.J.P.), taka.nojima@path.ox.ac.uk (T.N.)
https://doi.org/10.1016/j.molcel.2021.02.034

SUMMARY

Mammalian chromatin is the site of both RNA polymerase II (Pol II) transcription and coupled RNA processing. However, molecular details of such co-transcriptional mechanisms remain obscure, partly because of technical limitations in purifying authentic nascent transcripts. We present a new approach to characterize nascent RNA, called polymerase intact nascent transcript (POINT) technology. This three-pronged methodology maps nascent RNA 5′ ends (POINT-5), establishes the kinetics of co-transcriptional splicing patterns (POINT-nano), and profiles whole transcription units (POINT-seq). In particular, we show by depletion of the nuclear exonuclease Xrn2 that this activity acts selectively on cleaved 5′ P-RNA at polyadenylation sites. Furthermore, POINT-nano reveals that co-transcriptional splicing either occurs immediately after splice site transcription or is delayed until Pol II transcribes downstream sequences. Finally, we connect RNA cleavage and splicing with either premature or full-length transcript termination. We anticipate that POINT technology will afford full dissection of the complexity of co-transcriptional RNA processing.

INTRODUCTION

Transcripts synthesized by RNA polymerase II (Pol II) are extensively co-transcriptionally processed. First a cap structure (7meGppp) is added to the transcript 5′ end soon after its exit from the Pol II complex. This defines all Pol II transcription and is ultimately required for efficient mRNA export and protein translation. As Pol II transcribes into the gene body, introns are removed by splicing through assembly of the spliceosome complex, beginning with recognition of the intron 5′ splice site (SS) by U1 small nuclear RNA (snRNA)-protein complex (U1 snRNP). Once Pol II reaches the intron 3′ end, U2 snRNP identifies the intronic branchpoint and 3′ SS, followed by assembly of a further complex set of U snRNPs (U4, U5, and U6) and other associated protein factors. The spliceosome so formed reorganizes the intron into a ribozyme-like structure, leading to intron excision and ligation of upstream to downstream exons. This mechanism implies an intron definition model, whereby appearance of intronic splice signals leads to stepwise assembly of the spliceosome. However, in higher eukaryotes, in which exons are generally much shorter than adjacent introns, splicing factors such as SR proteins initially bind to and define functional exons, known as the exon definition model (Ule and Blencowe, 2019). This acts to recruit U2 snRNP to the 3′ SS and U1 snRNP to the 5′ SS, leading to spliceosome formation. At the gene end (polyadenylated transcript end site [TES]), specific polyadenylation sites (PAS) are recognized by the cleavage and polyadenylation (CPA) complex. An endonuclease CPSF73 within CPA complex cleaves the nascent RNA at the PAS, coupled with upstream RNA polyadenylation by polyA polymerase (Mandel et al., 2006). This promotes release of the mature mRNA from chromatin into the nucleoplasm and its subsequent transport to cytoplasmic ribosomes. The downstream RNA cleavage product is then degraded by the nuclear 5′-3′ exonuclease Xrn2, which follows behind elongating Pol II and induces transcription termination upon reaching the Pol II complex (Proudfoot, 2016). Nascent transcript cleavage also occurs in other transcript regions such as at pre-microRNA (miRNA) sequences. Here hairpin structures

This is an open access article under the CC BY license (http://creativecommons.org/licenses/by/4.0/).

are recognized by Microprocessor, a double-strand RNA (dsRNA)-specific endonuclease complex comprising dsRNA binding protein DGCR8 and dsRNA-specific endonuclease Drosha (Ha and Kim, 2014). Notably dsRNA cleavage in exons, but not in introns, promotes Pol II transcription termination. Likely splicing impedes termination in the intron otherwise induced by Drosha cleavage (Dhir et al., 2015).

These RNA processing and cleavage events are tightly regulated during synthesis of all Pol II transcription units (TUs) from the TSS (transcription start site) to the TES. Several nascent RNA analyses have indicated how these RNA processing events are coupled to transcription (Stark et al., 2019). For example, the method of transient transcript sequencing (TT-seq) provides information on the extent Pol II TUs (Schwalb et al., 2016). We have also used mammalian native elongating transcript sequencing (mNET-seq), showing that the S5P isoform of Pol II C-terminal domain (CTD) is associated with Pol II pausing on spliced exons and in recruiting the catalytic spliceosome (Nojima et al., 2015, 2018a; Schlackow et al., 2017). A critical limitation to both these methodologies is the restricted length of nascent transcript reads, which is limited to less than 150 nt because of RNA fragmentation in the protocols used, coupled with size limits set by the Illumina sequencing platform.

We describe new technology to dissect the complex Pol II transcription cycle by analyzing intact nascent RNA directly purified from elongating Pol II. This POINT (polymerase intact nascent transcript) methodology involves both Illumina and Oxford Nanopore Technologies (ONT) sequencing platforms. For Illumina we use *in vitro* fragmented RNA purified from immunoprecipitated Pol II elongation complexes to profile the nascent RNA across the whole TU (POINT-seq). We also use unfragmented RNA in a 5′ RACE-template switching protocol that maps nascent RNA 5′ ends at single-nucleotide resolution (POINT-5). Notably, POINT-5 precisely maps and distinguishes TSS and RNA cleavage sites on pre-mRNA, pre-miRNA, histone, and U snRNA genes. Xrn2-dependent RNA degradation at pre-mRNA TES is also detected by this technology. We further use the ONT direct cDNA sequencing platform to characterize nascent RNA isolated by POINT technology (POINT-nano), revealing the kinetics of splicing and CPA-mediated RNA cleavage.

RESULTS

Development of POINT technology

The analysis of authentic nascent RNA is critical to fully comprehend the mechanisms of Pol II transcription and associated transcript processing. POINT technology allows the isolation of intact nascent transcript from the 5′ cap site through to its 3′ end within the Pol II active site (Figure 1A). This involves chromatin solubilization by 1 M urea and 3% Empigen treatment and so allows efficient DNA specific digestion by DNase, which unlike MNase cannot access DNA in standard chromatin preparations. Pol II associated with its nascent transcript remains intact under these conditions. Notably, although the chromatin-associated DNA is fully digested to nucleosome size fragments within 4 min, a longer digestion period (12 min) more completely digests all DNA outside of the Pol II elongation complex. In this way, potential background proteins and RNA non-specifically associated with DNA external to Pol II are eliminated (Figures S1A–S1C). Following DNase digestion, intact nascent RNA ending within the Pol II active site is immunoprecipitated in the presence of 3% Empigen to remove steady-state mRNA and rRNA from the immunoprecipitated fraction (Figure S1D). We directly measured potential contamination in our POINT-derived RNA preparations by comparing POINT- with mNET-isolated RNA, as both methods involve RNA extraction from chromatin by Pol II immunoprecipitation (IP). Notably, while mNET-seq showed trace reads of contaminating tRNA and rRNA, POINT-seq showed even lower contamination levels (Figure S1E). The POINT-immunoprecipitated RNA, with a size range from a short length to greater than 6,000 nt, is either fragmented and sequenced (POINT-seq) or directly subjected to 5′ RACE and template switching (POINT-5) using the Illumina strand-specific RNA sequencing (RNA-seq) platform (Figure 1A).

We compared POINT-seq data with chromatin-bound RNA (ChrRNA-seq) data from HeLa cells (Nojima et al., 2015). ChrRNA-seq meta-profiles of protein coding (PC) genes show highest signal near the TES, followed by a sharp drop, indicative of substantial contamination by mRNA that have been cleaved at the TES and released into the nucleoplasm. In contrast, the POINT-seq profiles of PC genes detected highest signal over the TSS that gradually declined across the TU as well as higher signals in antisense and termination regions (Figure 1B). These differences underline the predominantly nascent transcription detected by POINT methodology. Additionally, transcriptional inhibition by 4 h treatment with DRB dramatically reduced POINT-seq signals over PC genes, including *TARS* (Figure S1F). Importantly, mature, exonic RNA was undetectable on POINT-seq from DRB-treated cells. These results confirm that POINT-seq profiles reflect highly purified nascent RNA, reproducible between three biological replicates (Figure S1G).

POINT-5 profiles allowed detection of all 5′ ends of non-overlapping PC genes expressed in HeLa cells, indicative of active TSS (Figure 1C) and show overall high data reproducibility (Figure S1H). We also used POINT-5 to characterize TSS from divergent TUs. This effectively maps 5′ ends of promoter-associated antisense transcripts (PROMPT), as shown for *KIAA0513* (Figure 1D) as well as divergent mRNA-mRNA and eRNA-eRNA pairs (Figures S1J and S1K). Next, we compared POINT-5 with published cap analysis of gene expression (CAGE) (Chen et al., 2016). CAGE detected robust signals for mRNA but little PROMPT signal. This increased upon depletion of the exosome component RRP40 (Figures 1E and 1F), as PROMPTs are known to be degraded by the exosome (Andersson and Sandelin, 2020; Preker et al., 2008; Schlackow et al., 2017). In contrast RRP40 depletion had no effect on CAGE signal from divergent mRNA (Figures 1G and 1H). Notably POINT-5 detected significant levels of PROMPT signal without exosome depletion (Figures 1E and 1F). This comparison of POINT-5 versus CAGE emphasizes the nascent nature of POINT-5 data and confirms that it provides a reliable approach to detect all categories of newly synthesized, capped Pol II transcripts.

POINT-5 defines co-transcriptional RNA cleavage sites

Nascent RNA can be cleaved during transcription by complexes containing endonucleases such as the Microprocessor

CellPress
OPEN ACCESS

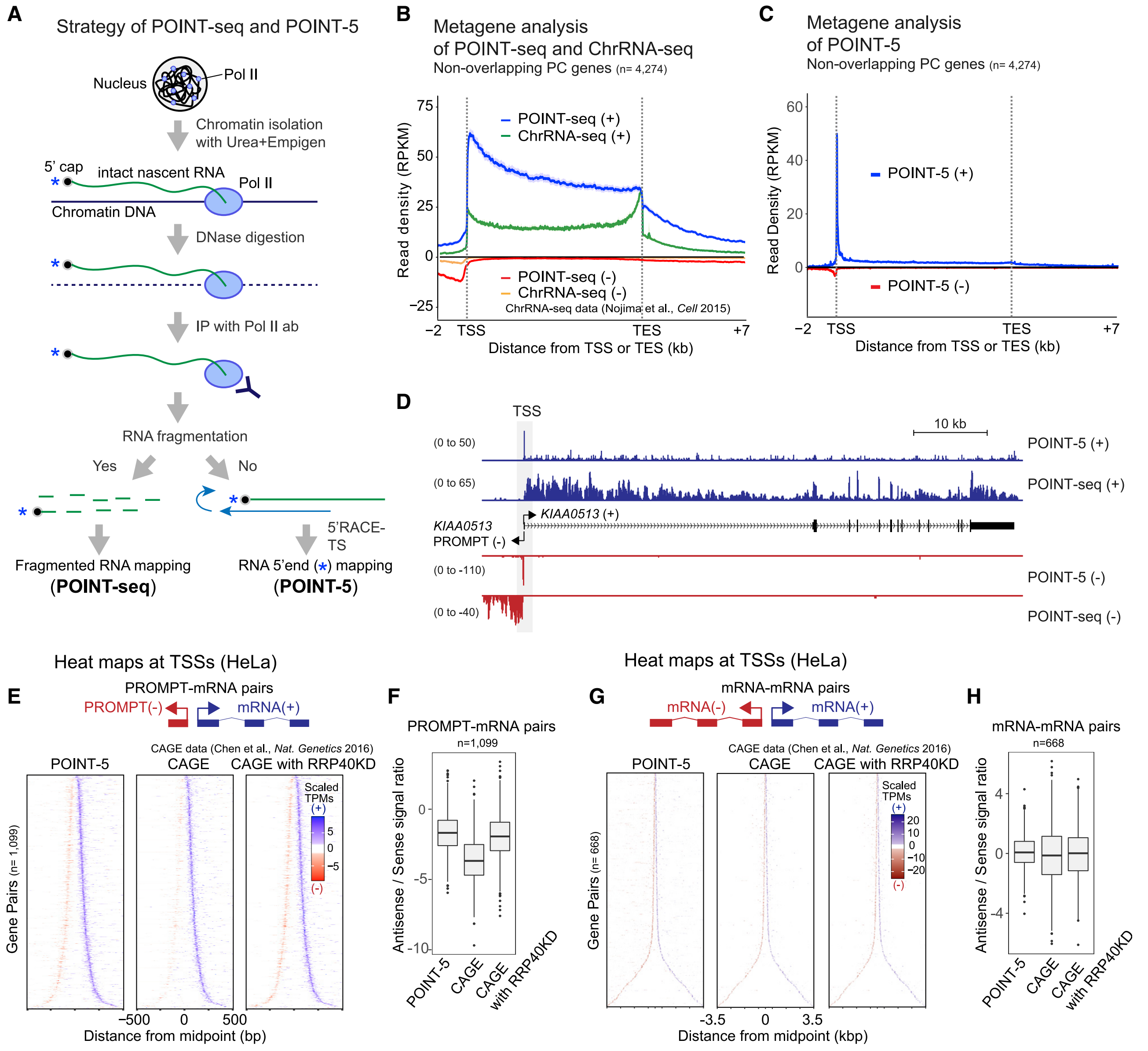

Figure 1. Development of POINT-seq and POINT-5 methodology

(A) Strategy of POINT technology. Chromatin fraction was digested with Turbo DNase. Intact nascent RNA immunoprecipitated with anti-Pol II antibody. For POINT-seq, RNA was fragmented during library preparation. For POINT-5, non-fragmentated RNA was reverse-transcribed with random hexamer and template switching during library preparation. Both POINT-seq and POINT-5 libraries were Illumina-sequenced.

(B) Metagene of POINT-seq and ChrRNA-seq signals in normalized transcription unit from TSS −2 kb to TES +7 kb of non-overlapping PC genes. Note that TSS and TES denote the major transcription start sites and PAS, respectively. POINT-seq profiles at (+) and (−) stands are shown in blue and red, respectively. Published chromatin RNA (ChrRNA)-seq profiles at (+) and (−) stands are shown in green and orange.

(C) Metagene of POINT-5 signals in normalized transcription unit from TSS −2 kb to TES +7 kb of non-overlapping PC genes. POINT-5 profiles at (+) and (−) stands are shown in blue and red.

(D) *KIAA0513* as an example of POINT-5 and POINT-seq profiles.

(E) Heatmaps of POINT-5 and CAGE (untreated or RRP40KD) signals for PROMPT-mRNA pairs at TSS. KD, knockdown or depletion. Scaled transcripts per million (TPM) is shown for sense (+) (blue) and antisense (−) (red).

(F) Quantitation of (E).

(G) Heatmaps of POINT-5 and CAGE (untreated or RRP40KD) signals for mRNA-mRNA pairs at TSS. Scaled TPM is shown for sense (+) (blue) and antisense (−) (red).

(H) Quantitation of (G).

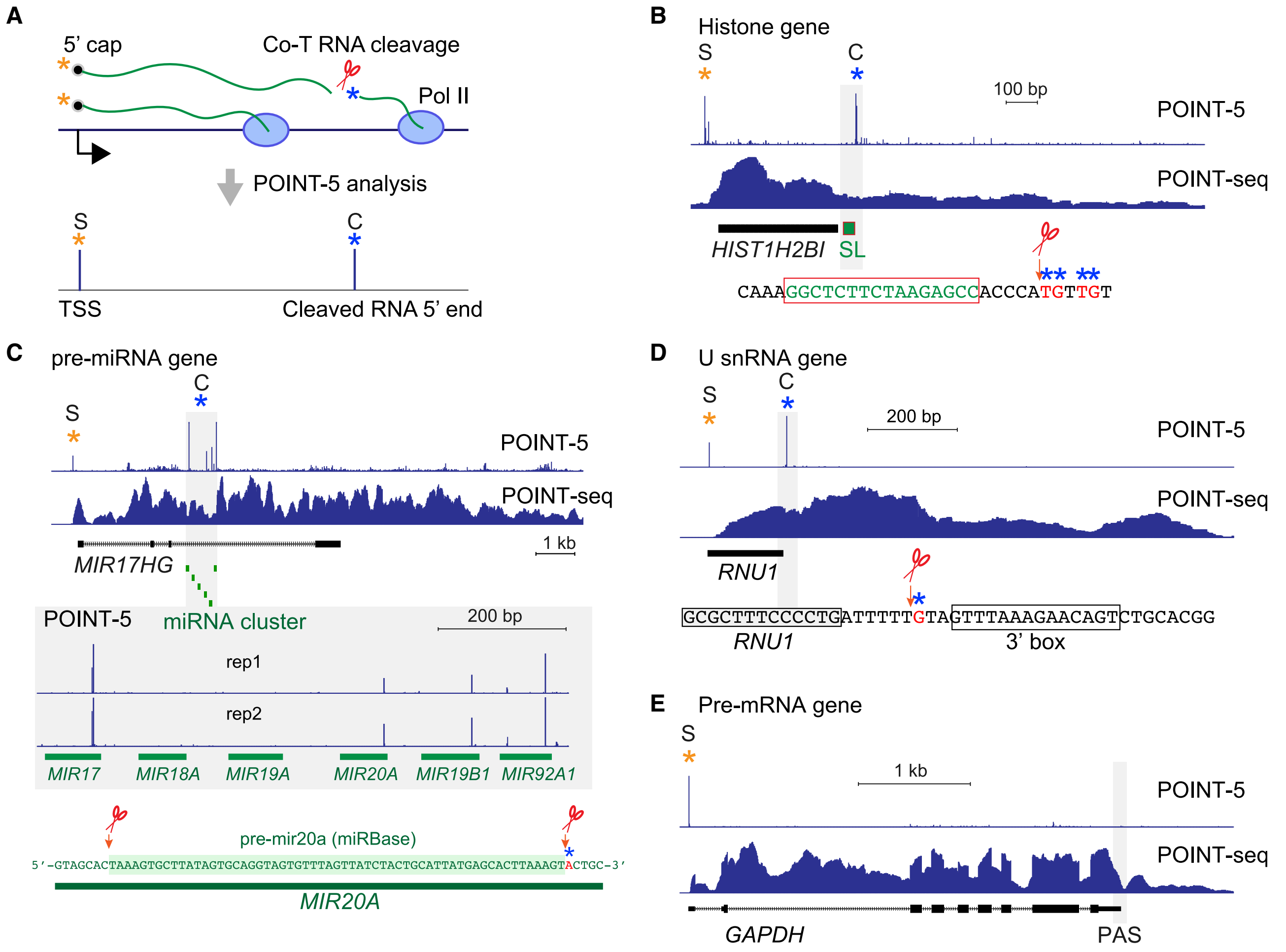

Figure 2. Detection of nascent RNA 5′ ends by POINT-5

(A) Schematic diagram of POINT-5 analysis detecting TSS (S, orange asterisk) and co-transcriptional (co-T) RNA cleavage site (C, blue asterisk).

(B) Histone gene: *HIST1H2BI* POINT-5 and POINT-seq profiles. Stem loop (SL) in green.

(C) Pre-miRNA gene: *MIR17HG* POINT-5 and POINT-seq profiles. Cluster of miRNA shown in green. POINT-5 peak detected near 3′ end of *MIR20A* as previously reported in miRBase.

(D) U snRNA gene: *RNU1* POINT-5 and POINT-seq profiles. Co-T RNA cleavage was detected upstream of 3′ box.

(E) Pre-mRNA gene: *GAPDH* POINT-5 and POINT-seq profiles. No POINT-5 peak was detected at TES.

(Morlando et al., 2008; Nojima et al., 2015; Pawlicki and Steitz, 2008). Also, the U7 snRNA-CPA complex cleaves histone transcripts following their downstream stem loop (SL) structure (Marzluff and Koreski, 2017), while the Integrator complex is known to terminate U snRNA gene transcription (Chen and Wagner, 2010). Each complex possesses endonuclease activity, thought to act co-transcriptionally. Notably, POINT-5 methodology can map co-transcriptional (co-T) RNA cleavage sites at single-nucleotide resolution, as with the TSS, by detecting newly synthesized 5′ ends of nascent RNA associated with the Pol II active site (Figure 2A).

We first applied POINT-5 technology to define endonuclease cleavage positions across a human histone gene locus (Figure S2A). Clear peaks were detected at either end of each histone gene, one at the TSS (S peak) and another resulting from endonuclease cleavage (C peak). With HIST1H2BI, the C peak showed local heterogeneity over several nucleotides, downstream of the SL structure that defines the 3′ end of histone mRNA (Figure 2B). POINT-5 analysis also detected significant peaks indicative of Drosha cleavage activity over intronic MIR26B in *CTDSP1* and long noncoding RNA (lncRNA)-derived MIR193A and MIR365B (Figures S2B and S2C). Notably 3′ ends of the co-transcriptionally cleaved pre-miRNA cluster MIR17-92A, embedded within the lncRNA *MIR17HG*, were previously detected using mNET-seq (Nojima et al., 2015). Our POINT-5 method instead maps the 5′ end of these Drosha cleaved RNAs associated with Pol II. Published ENCODE databases show that all six miRNAs are expressed from this cluster, while POINT-5 and mNET-seq data detect only co-transcriptionally cleaved 5′ and 3′ ends for MIR17, 20a, 19b-1, and 92a-1 (Figure 2C). This suggests that pre-miR18a and 19a are cleaved post-transcriptionally.

Transcription termination of U snRNA genes is known to be induced by the Integrator complex, which recognizes a

CellPress
OPEN ACCESS

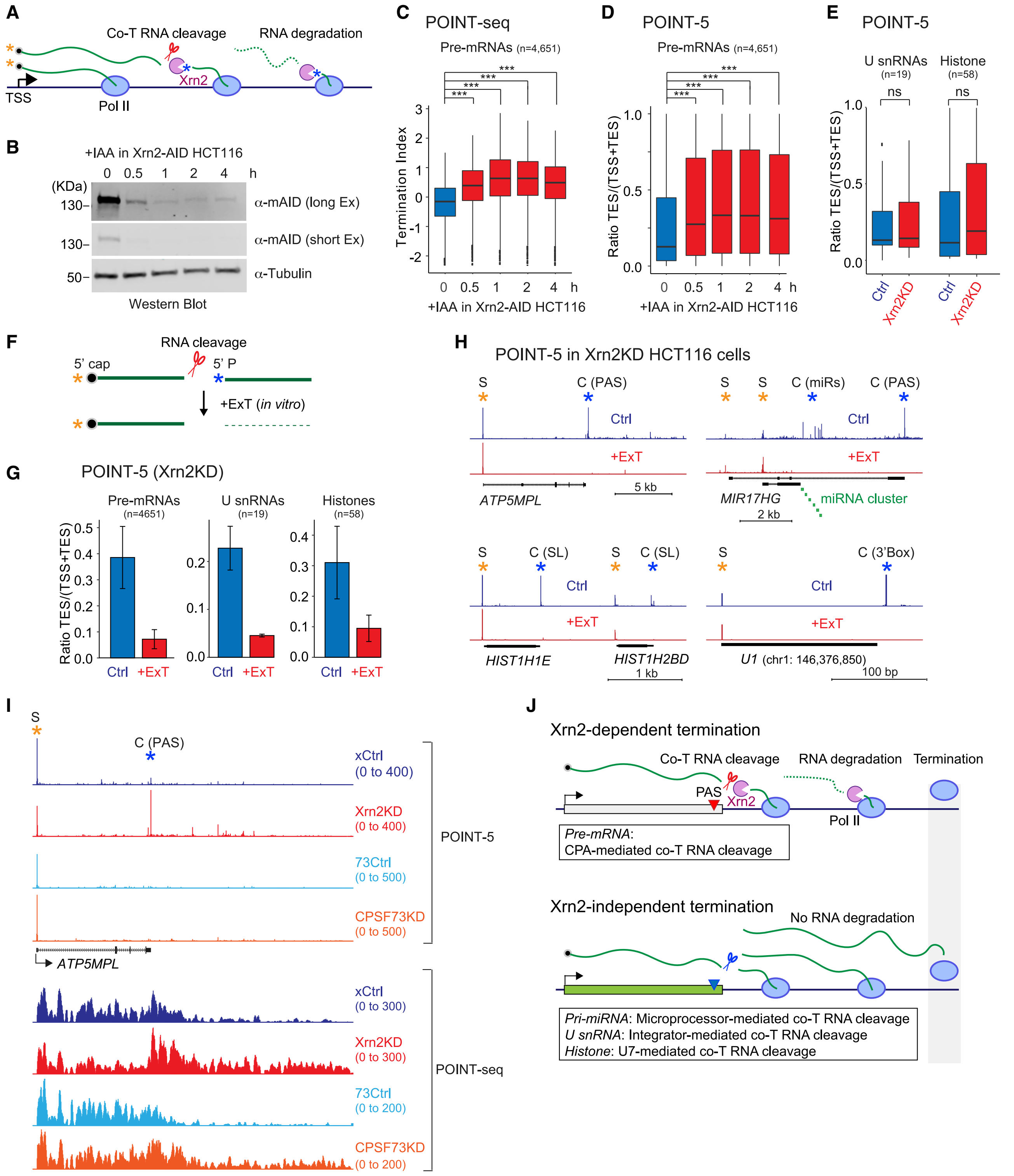

Figure 3. Specificity of Xrn2 exonuclease

(A) Schematic of co-T RNA cleavage and degradation.

(B) Western blot of Xrn2-AID: time course of IAA treatment in HCT116 Xrn2-AID cells (two exposures). Anti-tubulin antibody used as loading control.

(C) Termination index. Bar plots of POINT-seq signal ratio of gene body and termination region upon Xrn2 depletion (KD) time course for non-overlapping protein coding (PC) genes expressed in HCT116 Xrn2-AID cells. Without (blue) or with (red) Xrn2KD.

(legend continued on next page)

downstream consensus element (3′ box) and cleaves at adjacent RNA sequence (Chen and Wagner, 2010). Although RNA cleavage sites of transfected or *in vitro* synthesized U snRNA were previously characterized using an RNase protection assay (Uguen and Murphy, 2003), POINT-5 analysis shows endogenous co-T RNA cleavage on U snRNA genes with C peaks, presumably derived from Integrator-associated endonuclease activity (known to be present in the IntS11 subunit). This cleaves pre-U snRNAs immediately upstream of the consensus 3′ box sequence (Figures 2D and S2D). It is evident for these small Pol II TUs that 3′ processing occurs well before the end of the snRNA primary transcript.

As shown above, strong C peaks were detected for histone gene transcripts, pre-miRNA, and U snRNA, even though derived from different co-T RNA cleavage activities. Instead, we fail to detect C peaks for pre-mRNA PAS. Thus, for *GAPDH*, significant signal was detected only at the TSS (S peak), not at the TES (C peak), although endonuclease cleavage at PAS by CPSF73 is well established (Figure 2E). Likely 5′-3′ exonuclease Xrn2 selectively and rapidly degrades downstream RNA following cleavage at the PAS, but not from the other endonuclease cleavage sites, as addressed below.

Specific Xrn2-dependent transcription termination of PC genes

Transcription termination of PC genes is associated with degradation of downstream RNA by the nuclear 5′-3′ exonuclease Xrn2, following 3′ end CPA of the pre-mRNA (Proudfoot, 2016; West et al., 2004) (Figure 3A). The role of this enzyme in processing TES-associated transcripts was previously investigated by degron (AID) tagging of endogenous Xrn2 in HCT116 cells also engineered to express the plant TIR gene, activated by auxin (IAA) treatment to degrade Xrn2-AID (Eaton et al., 2018). We have used this Xrn2 degron cell line in our POINT-5 analyses. Note that the AID tag renders Xrn2 unstable even without IAA treatment, so that these control cells (Ctrl HCT116) generate substantially less Xrn2 protein than either wild-type HCT116 (Eaton et al., 2018) or HeLa cells (Figure S3A). Furthermore, near complete depletion (knockdown [KD]) of Xrn2-AID protein was induced by IAA treatment over 4 h (Figures 3B). Consistent with previous analysis using mNET-seq (Eaton et al., 2018), POINT-seq shows that rapid depletion of Xrn2 protein induced immediate Pol II termination defects on PC genes, with RNA accumulating downstream of the PAS (Figures 3C and S3B). Similarly POINT-5 detected higher signal at TES regions of PC genes following Xrn2 depletion (Figures 3D and S3C). Note that Ctrl HCT116 cells generally show a clear TES peak (Figures S3C and S3D) in contrast to HeLa cells, in which TES peaks are barely detected (Figure 1C) because of higher levels of Xrn2 in HeLa cells than Ctrl HCT116 cells (Figure S3A). Both defects in transcription termination and reduced RNA degradation at PAS were observed from 30 min of IAA treatment (Figures 3C and 3D), suggesting direct effects of Xrn2 protein. Xrn2 depletion did not affect the C peaks of either histone and or U snRNA genes, as shown by POINT-5 analysis (Figures 3E and S3E). In addition, POINT-5 C peaks at intronic pre-miRNAs (MIR17HG cluster) were unaffected by Xrn2 depletion, although the C peak of the host gene PAS was significantly increased (Figure S3F). Notably, no effect of Xrn2 depletion on intergenic pre-miRNA (MIR331) was detected, even though the pre-miRNA is located downstream of the host gene *VEZT* PAS (Figure S3F). These results underline the specificity of Xrn2 for PAS mediated cleavage.

Overall the above results indicate that rapid Xrn2 depletion causes termination defects following CPA cleavage of PAS. As POINT-5 methodology detects all nascent RNA 5′ ends irrespective of their chemical nature, we next sought to categorize POINT-5 signals into either 5′-capped or uncapped 5′ monophosphate (5′ P)-RNA. In particular, Xrn2 exonuclease activity is specific for 5′P-RNA substrates. We therefore used the 5′ P-RNA-specific nuclease (ExoTerminator [ExT]) to *in vitro* digest immunoprecipitated nascent RNA fractions, as isolated using POINT technology. This will selectively degrade Xrn2-sensitive 5′ P-RNAs (Figure 3F). Notably POINT-5 TES signals for polyA+ pre-mRNA, U snRNA, and histone pre-mRNA were all substantially reduced by *in vitro* ExT treatment, but not TSS-associated S signals (Figures 3G, 3H, and S3G). This confirms that the POINT-5 TES signals, sensitive to ExT, derive from RNA cleavage and not from alternative TSS. For example, POINT-5 peaks downstream to *JARID2* TSS2 are ExT sensitive, but not Xrn2 dependent, suggesting that PAS-independent RNA cleavage occurs near this alternative TSS of *JARID2* (Figure S3G).

Another feature of rapid Xrn2-AID depletion was the appearance of multiple POINT-5 peaks situated downstream of PAS that all display ExT sensitivity (Figures 3I and S3G). These are either intermediates of Xrn2-mediated RNA degradation or RNA cleavage sites of other RNA endonucleases such as Integrator or Drosha that do not induce Xrn2-dependent RNA degradation. We next induced defective transcription termination by CPSF73 degron-mediated depletion (Eaton et al., 2020) and determined whether POINT-5 peaks located downstream of PAS are affected. CPSF73 depleted by IAA treatment of HCT116 CPSF73-AID cells for 3 h (Figure S3H) showed clear termination defects for non-overlapping PC genes by POINT-seq analysis (Figure S3I). However, no POINT-5 peaks were detected at the PAS, even in control cells, because of high Xrn2 protein levels in HCT116 CPSF73-AID cells (Figure S3J). Notably CPSF73 depletion did not induce POINT-5 peaks downstream of

(D) Bar plots of POINT-5 signal ratio of TSS and TES upon Xrn2KD time course for non-overlapping PC genes expressed in HCT116 Xrn2-AID cells as in (C).
(E) Bar plots of POINT-5 signal ratio of TSS and TES upon Xrn2KD (4 h) for U snRNA and histone genes expressed in HCT116 Xrn2-AID cells as in (C).
(F) Schematic diagram of ExoTerminator (ExT) treatment. Uncapped 5′ P-RNA generated by RNA cleavage and specifically degraded by *in vitro* ExT treatment.
(G) Bar plots of POINT-5 signal ratio of TSS and TES upon Xrn2KD (4 h) for non-overlapping PC genes, U snRNA, and histone genes expressed in HCT116 Xrn2-AID cells. Without (blue) or with (red) ExT treatment.
(H) Examples of POINT-5 upon Xrn2KD (4 h) with ExT treatment in HCT116 Xrn2-AID cells.
(I) POINT-seq and POINT-5 of *ATP5MPL* in Xrn2KD (4 h) or CPSF73KD (3 h) or control (Ctrl) HCT116 cells.
(J) Model for Xrn2-dependent or independent termination mechanisms.

CellPress
OPEN ACCESS

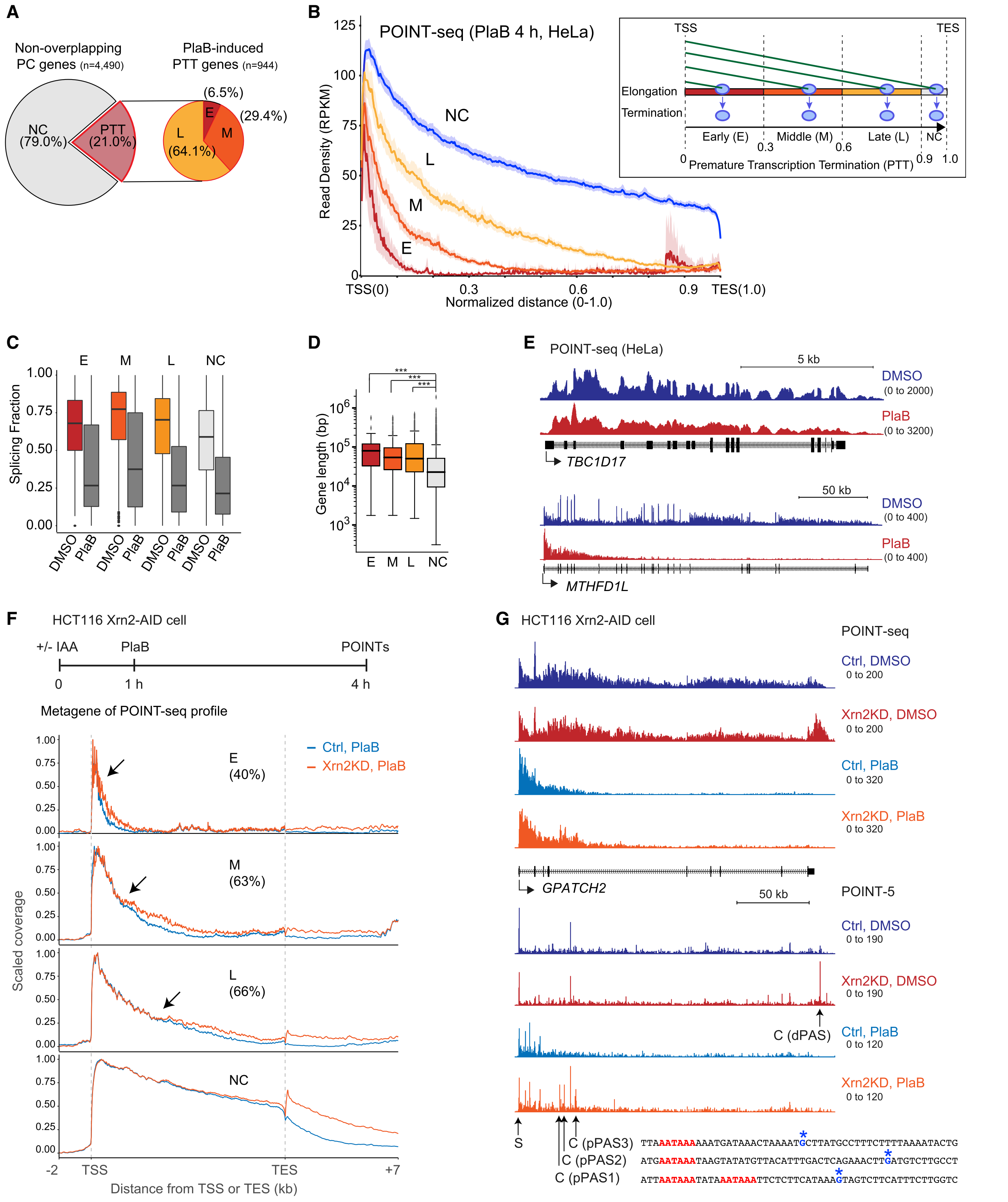

(legend on next page)

ATP5MPL PAS (Figure 3J), suggesting that Xrn2-independent RNA degradation does not occur in this termination region. This further indicates that Xrn2 depletion-dependent peaks at the termination site are intermediates of Xrn2-mediated RNA degradation or CPA-cleaved RNA downstream of functional PAS.

Our POINT technology reveals the precise locations of co-T RNA cleavage sites and also their Xrn2 dependency. PC RNA (pre-mRNA) is cleaved by CPA complex at the PAS and then Xrn2-degraded toward elongating Pol II, resulting in transcription termination (Figure 3J). In contrast, histone pre-mRNA, U snRNA, and pri-miRNA are cleaved in a CPA-independent manner. Transcription termination of such genes appears Xrn2 independent, indicating that their 3′ end cleaved RNA is undegraded. Possibly these RNA cleavage activities trigger different termination mechanisms such as transcription roadblock effects caused by loss of transcription elongation factors, DNA structures, or higher nucleosome density (Proudfoot, 2016).

Splicing suppresses premature transcriptional termination

Cryptic, inactive PAS are often embedded within mammalian introns, where their use may be restricted by limiting levels of CPA factors or more efficient Pol II elongation (Kamieniarz-Gdula and Proudfoot, 2019). Furthermore, U1 antisense morpholino (AMO) that blocks U snRNA base pairing with 5′ SS often leads to activation of intronic PAS and consequent premature transcriptional termination (PTT) (Kaida et al., 2010; Oh et al., 2017). The specificity of this effect (called telescripting) was implied by use of U2 AMO, which although inhibiting splicing did not induce PTT in a selected gene normally affected by U1 AMO (Oh et al., 2017). Recently U1 snRNA bound to 5′ SS has been shown to inhibit CPA complex activity and so suppress PTT by preventing nearby PAS recognition (So et al., 2019). However, it remains a possibility that splicing may more generally prevent PTT.

We determined the effect of pladienolide B (PlaB) treatment of HeLa cells on our POINT-seq profiles, as this inhibitor targets the SF3B complex, a component of U2 snRNP (Kotake et al., 2007) and suppresses co-T splicing (Nojima et al., 2015). Unexpectedly PlaB induced PTT in 21% of PC genes expressed in HeLa cells, which we divided into three gene regions (Figure 4A); early (E), middle (M), and late (L) of each TU (Figure 4B). As expected, PlaB treatment induced global splicing inhibition (Figure 4C). Notably, PTT induced by PlaB preferentially occurred in long genes (Figure 4D), presumably because Pol II will encounter more cryptic PAS in longer TUs, as illustrated for *TBC1D17*, showing only splicing inhibition and for much longer *MTHFD1L* with both splicing and PTT effects induced by PlaB treatment (Figure 4E). Comparison of databases from HeLa cells treated with U1 AMO for 8 h (Oh et al., 2017) with our POINT-seq data reveals that some genes display PTT by both U1 AMO and PlaB treatments (Figure S4A) as for *MTHFD1L* (Figure S4B). In contrast, *DHX9* shows a clear U1 AMO effect but does not display PTT effects following PlaB treatment (Figure S4B). The fact that telescripting and general splicing inhibition by PlaB treatment can often affect different genes may indicate different cryptic PAS sensitivities.

To establish that PlaB-induced PTT is regulated by cryptic PAS activation, we depleted either Xrn2 or CPSF73 proteins for 1 h, followed by PlaB treatment for 3 h in the AID-engineered HCT116 cells. POINT-seq analysis reveals that PTT transcripts are indeed extended as shown in metagene profiles (Figures 4F and S4C) and for *GPATCH2* (Figures 4G and S4D), indicating that ~60% of the PTT cases are PAS dependent. Furthermore, Xrn2 depletion followed by PlaB treatment induced POINT-5 peaks at multiple cryptic PAS (pPAS1–3) in intron 4 of *GPATCH2* (Figure 4G). We also detected Xrn2-independent POINT-5 peaks with PlaB-treated cells (Figure 4G; intron 1 of *GPATCH2*). This implies that CPSF73-independent RNA cleavage and Xrn2-independent RNA degradation may also be involved in premature termination.

POINT-nano methodology profiles single-molecule nascent transcripts

Technical issues have hitherto limited a full mechanistic understanding of co-T RNA processing. Illumina sequencing can be used to map short nascent RNA fragments, either metabolically labeled by 4sU (Schwalb et al., 2016) or isolated from within Pol II elongation complexes (Nojima et al., 2015). However, read lengths of individual RNA species were generally too short to determine the kinetics of co-T splicing, interwoven with other RNA processing mechanisms. Indeed, our previous mNET-seq analysis of spliced RNA associated with the Pol II active site was limited to investigation of short exons in which immediate upstream splicing was still detected (Nojima et al., 2018a). We have now used longer read ONT sequencing on full-length nascent transcripts isolated using POINT technology (POINT-nano; Figure 5A). This RNA was size-selected (longer than 500 nt) to avoid short reads in ONT sequencing and

Figure 4. Suppression of PTT by splicing

(A) Pie charts for fractions of premature transcription termination (PTT) in non-overlapping genes, induced by PlaB. No change (NC), PTT; early PTT (E), middle PTT (M), and late PTT (L).

(B) Four classes (E, M, L, and NC) of PTT induced by PlaB treatment for 4 h. Metagene of POINT-seq in PlaB-treated HeLa cells (left). Diagram of PTT in four different regions of normalized gene (right).

(C) Splicing fraction in four classes of PTT in genes inhibited by PlaB.

(D) Gene length in four classes of PTT in genes induced by PlaB.

(E) Examples of POINT-seq signals on *TBC1D17* (short gene) and *MTHFD1L* (long gene) in HeLa cells.

(F) Top: schematic protocol of Xrn2KD and PlaB treatment in HCT116 Xrn2-AID cells. Bottom: metagene profile of POINT-seq for four classes (E, M, L, and NC) with PlaB (light blue) or Xrn2KD and PlaB (orange) in normalized region from TSS −2 kb to TES +7 kb. Percentages of PTT gene cases recovered by Xrn2KD are shown. Arrows indicate start positions of PTT defects induced by Xrn2KD. Percentages of PTT genes recovered by Xrn2KD are shown.

(G) POINT-seq and POINT-5 analyses of *GPATCH2* for indicated treatments. Distal (d)PAS and three cryptic proximal (p)PASs detected by POINT-5 under Xrn2KD and Xrn2KD + PlaB conditions, respectively.

CellPress
OPEN ACCESS

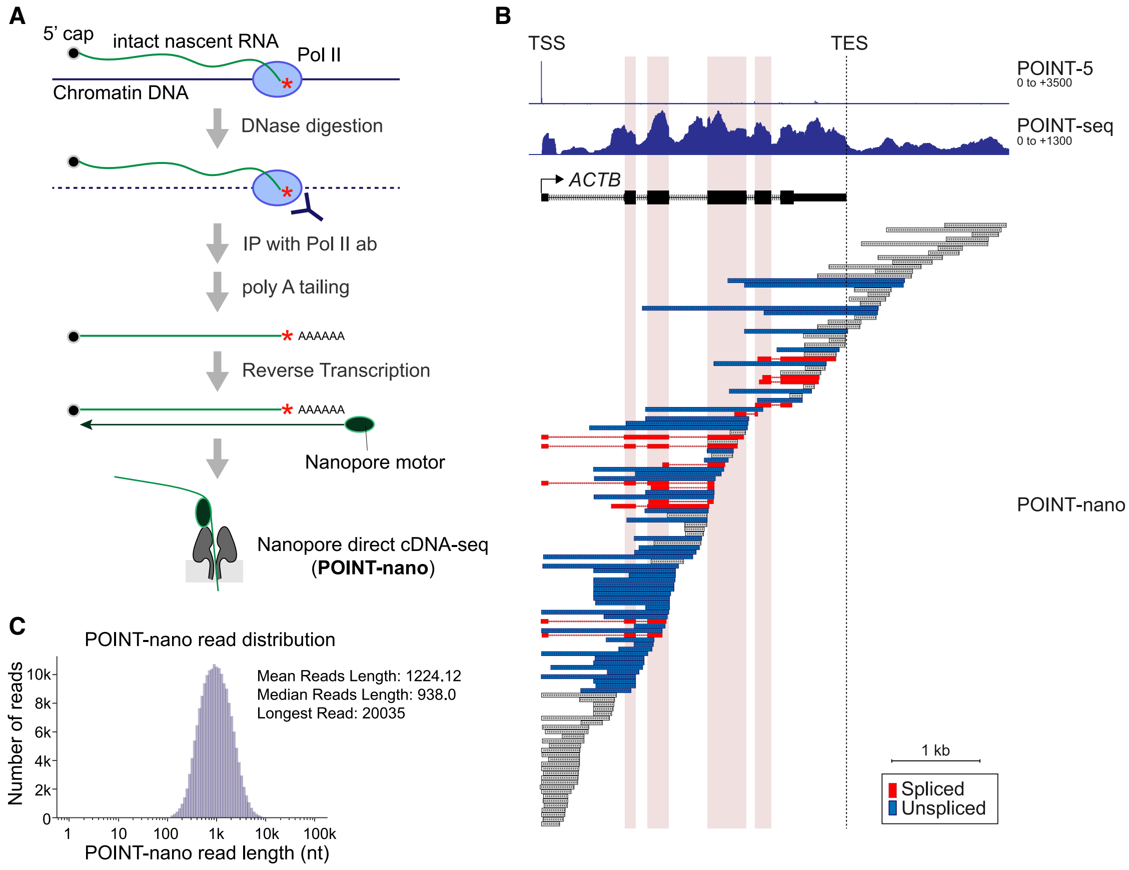

Figure 5. Development of POINT-nano methodology

(A) POINT-nano scheme. Intact nascent RNA was isolated as in other POINT technologies and *in vitro* polyadenylated. ONT direct cDNA sequencing was applied to polyA tailed intact nascent RNA.

(B) POINT-nano reads for *ACTB* with profiles of POINT-5 and POINT-seq also shown. Internal exons highlighted in pink. TES marked with dashed line. Spliced and unspliced POINT-nano reads indicated in red and blue, respectively. Other POINT-nano reads in gray.

(C) Distribution of POINT-nano reads.

subjected to *in vitro* 3′ end polyadenylation using bacterial-derived polyA polymerase (Figure S5A). Following reverse transcription with nanopore oligodT primers attached to a motor protein, single-molecule cDNA sequencing was performed using the high-throughput ONT device PromethION. Bioinformatic analysis of POINT-nano required prior removal of reads derived from oligodT priming on internal A-rich sequence (Figures S5B and S5C).

POINT-nano datasets are reproducible (Figure S5D), with mean coverage per non-overlapping PC gene of approximately 35 reads (Figure S5E). Comparing RNA 3′ end distributions for POINT-nano with mNET-seq databases across PC gene TUs shows a higher fraction of POINT-nano reads at the TES (Figure S5F). This may indicate that cleaved transcripts remain transiently tethered to elongating Pol II, as also recently observed (Drexler et al., 2020). POINT-nano reads are shown for *ACTB*, *ID1*, and *MAT2A* compared with POINT-5 and POINT-seq profiles (Figures 5B, S5G, and S5H). These align with PROMPTs and unspliced pre-mRNA (see *ID1*; Figure S5G), confirming that nascent transcripts are effectively captured by this methodology. A limitation to our POINT-nano technology is that read lengths are on average 1,224 nt (Figure 5C), while the intact nascent RNAs have a mean length of ~6,000 nt (Figure S5A). Even so, both spliced and unspliced reads are evident on most genes as shown for *ACTB* (Figure 5B). It is also difficult to determine whether read 5′ ends correspond to authentic TSS, TES, or *in vivo* degradation versus artificial read ends. However, multiple reads ending at the TSS as defined by the POINT-5 signal likely correspond to capped TSS transcript, while short reads 3′ to the TES may correspond to authentic 3′ processing by CPA with subsequent Xrn2 degradation. Thus, POINT-nano technology profiles the status of RNA processing and Pol II position in single RNA molecules, providing new insight into the kinetics of co-T processing.

POINT-nano identifies immediate and delayed co-T splicing

To determine the timing of pre-mRNA splicing relative to transcription, spliced and unspliced reads detected by POINT-nano sequencing were grouped according to their 3′ end position, in either exons or introns (Figure 6A). Forty percent of reads are already spliced as Pol II transcribes into an exon (Figure 6B),

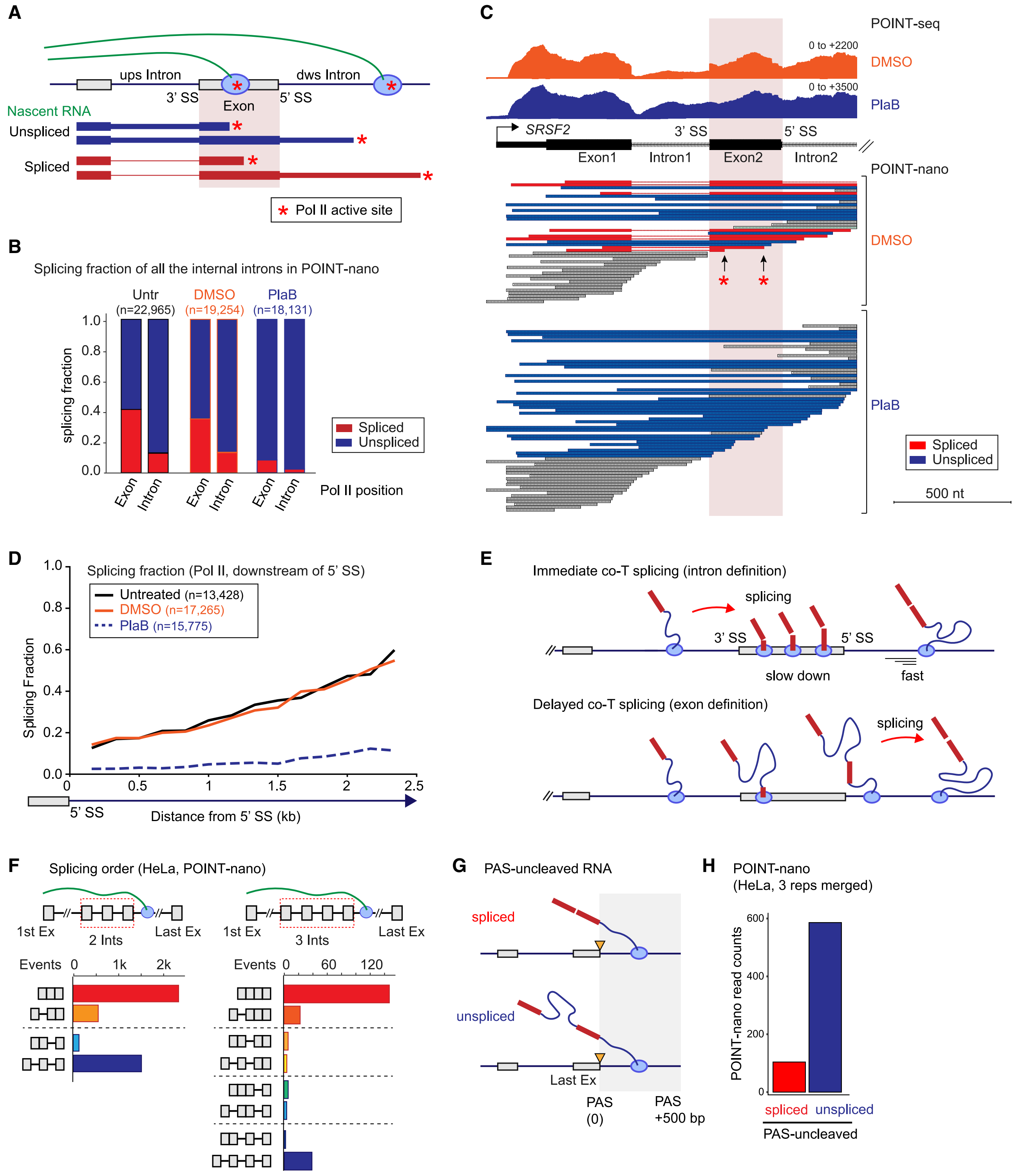

Figure 6. Two modes of co-T splicing in human cells

(A) Schematic of POINT-nano analysis on timing of co-T splicing. Splicing of downstream exon highlighted in pink. Spliced and unspliced POINT-nano reads indicated in red and blue, respectively. Pol II active site indicated by red asterisk.

(B) Splicing fraction of POINT-nano reads for all internal introns expressed in untreated (Untr), DMSO-treated, and PlaB-treated HeLa cells. Pol II positions indicated below.

(legend continued on next page)

CellPress
OPEN ACCESS

indicative of splicing by intron definition. Notably, some spliced reads were detected as early as 15 nt downstream of the 3′ SS (Figure S6A), consistent with the number of newly synthesized nucleotides within the Pol II complex (Martinez-Rucobo et al., 2015). These data reveal that splicing can occur as soon as the nascent transcript emerges from Pol II, as previously described for yeast splicing (Herzel et al., 2018; Oesterreich et al., 2016). This immediate splicing is readily detectible over specific genes. Thus the 5′ portion of *SRSF2* shows POINT-nano reads in which Pol II in exon 2 has already spliced out intron 1 (Figure 6C, red asterisks). Similarly, both *ACTB* and *MAT2A* (Figures 5B and S5H) show multiple examples of immediate splicing with several cases of mixed immediate and delayed splicing evident. As expected, the proportion of spliced POINT-nano reads was greatly reduced by PlaB treatment (Figures 6C, S6A, and S6B).

We also compared the transition between exons and downstream introns (Figure S6B). This shows that following higher levels of exon associated spliced reads because of immediate splicing, there are fewer spliced reads associated with Pol II positioned in the downstream intron. This striking positional difference is not due to selective read loss, as unspliced reads were detectible at equivalent levels for Pol II in both exons and adjacent intron sequence. Likely, the prevalence of spliced reads associated with exonic Pol II reflects a splicing-dependent reduction in elongation rate associated with assembly of the spliceosome (Nojima et al., 2018a). After immediate splicing and spliceosome disassembly, Pol II elongates faster in the downstream intron, with reduced density of spliced reads. In contrast, for delayed splicing, spliceosome components are not immediately recruited, so that Pol II elongation rates will be equivalent for both exon and intron, until eventually spliceosomes form. It is possible that unlike immediately spliced transcripts, unspliced transcripts associated with delayed splicing form R-loop structures with DNA duplex behind the elongating Pol II (Bonnet et al., 2017). This may contribute to the higher unspliced reads observed over intron sequence. Next, we analyzed the proportion of nascent transcripts that become spliced as Pol II transcribes further into downstream introns. To circumvent bias introduced by read length, the analysis focused on 5′ SS proximal sequence. Notably the proportion of spliced reads increased as Pol II transcribes downstream of the 5′ SS (Figure 6D). Overall, these POINT-nano data indicate that newly synthesized pre-mRNA is either immediately spliced following 3′ SS transcription (while Pol II is located in the exon), or splicing is delayed until Pol II transcribes ~2 kb of the downstream intron. We model these results to show how immediate or delayed co-T splicing reflects intron or exon definition mechanisms, respectively (Figure 6E).

We further investigated the ability of POINT technology to discriminate differences in SS strength. The proportion of spliced reads aligned with weak SSs was significantly lower compared with reads spanning strong SSs (Figures S6C and S6D). As alternatively spliced exons have weaker SSs (Itoh et al., 2004), we determined whether POINT technology detects differences in co-T splicing efficiency between constitutive splicing (CS) and alternative splicing (AS) events. We used POINT-seq data to show that the proportion of spliced reads is significantly higher in CS exons compared with AS exons (Figure S6E). To determine the timing of AS, we focused on cassette exon splicing events. These were classified into high or low exon inclusion categories using previously published pA+ RNA-seq data (Figure S6F). As expected, our POINT-nano data reiterated this classification. Importantly, whether or not the internal cassette exon was included or excluded, splicing levels of external exons were maintained (Figure S6G), suggesting that either events can be regulated by immediate splicing (i.e., intron definition). However, it is possible that cassette exon splicing is also regulated by delayed splicing (i.e., exon definition), as POINT-nano data have limited read length.

We next used POINT-nano to investigate splicing order along the TU by analysis of the order of excision of either two or three internal introns (Figure 6F). Mostly all introns were excised (red bars). However, sometimes an upstream intron was left unspliced, even though the downstream intron was excised (orange bars), arguing that splicing does not always occur sequentially. A subset of transcripts was also observed with all introns unspliced (dark blue bars). We next analyzed only genes for which full-length reads were obtained using POINT-nano (i.e., genes with TUs shorter than 1,500 nt). Full-length reads ending at the TES were detected in 10 genes, which were mainly fully spliced (Figure S6H). In contrast, full-length reads ending past the TES were observed in more than 240 genes and were mostly fully unspliced (Figure S6H). To further investigate the timing of splicing relative to 3′ end CPA, we analyzed all POINT-nano reads with 3′ ends located up to 500 nt past the TES (Figure 6G). Multiple unspliced reads corresponding to nascent transcripts not cleaved at the PAS were observed (Figure 6H). Consistently, we show reduced splicing of the last intron with Pol II beyond the PAS compared with when it is in the last exon (Figure S6I). Whether such transcripts are eventually processed and exported to the cytoplasm or targeted for degradation in the nucleus remains to be established.

Extending TU size enhances co-T splicing

We observe that the proportion of spliced reads gradually increase as Pol II transcribes across introns (Figure 6D), indicating that intron length enhances splicing. Exons and introns were size-classified into either long (top 25%) or short (bottom 25%), and the proportion of spliced POINT-nano reads was determined in these two categories. Notably splicing levels

(C) POINT-nano reads of *SRSF2* 5' side with profiles of POINT-seq in DMSO and PlaB-treated HeLa cells. Spliced and unspliced POINT-nano reads indicated in red and blue, respectively. Other POINT-nano reads in gray. Pol II active sites on spliced exon 2 indicated as red asterisks.

(D) Splicing fraction of POINT-nano reads with Pol II located in downstream of intron. Untreated, DMSO-treated, and PlaB-treated HeLa cell shown as black, orange, and blue dashed lines, respectively.

(E) Immediate and delayed co-T splicing models.

(F) Splicing order analysis of POINT-nano reads for (left) two and (right) three internal introns in HeLa cells.

(G) Diagram illustrating spliced/unspliced PAS-uncleaved transcripts over TES region (PAS + ~500 nt).

(H) Splicing fraction for last intron removal of PAS-uncleaved RNA over TES region (PAS + ~500 nt) in POINT-nano analysis.

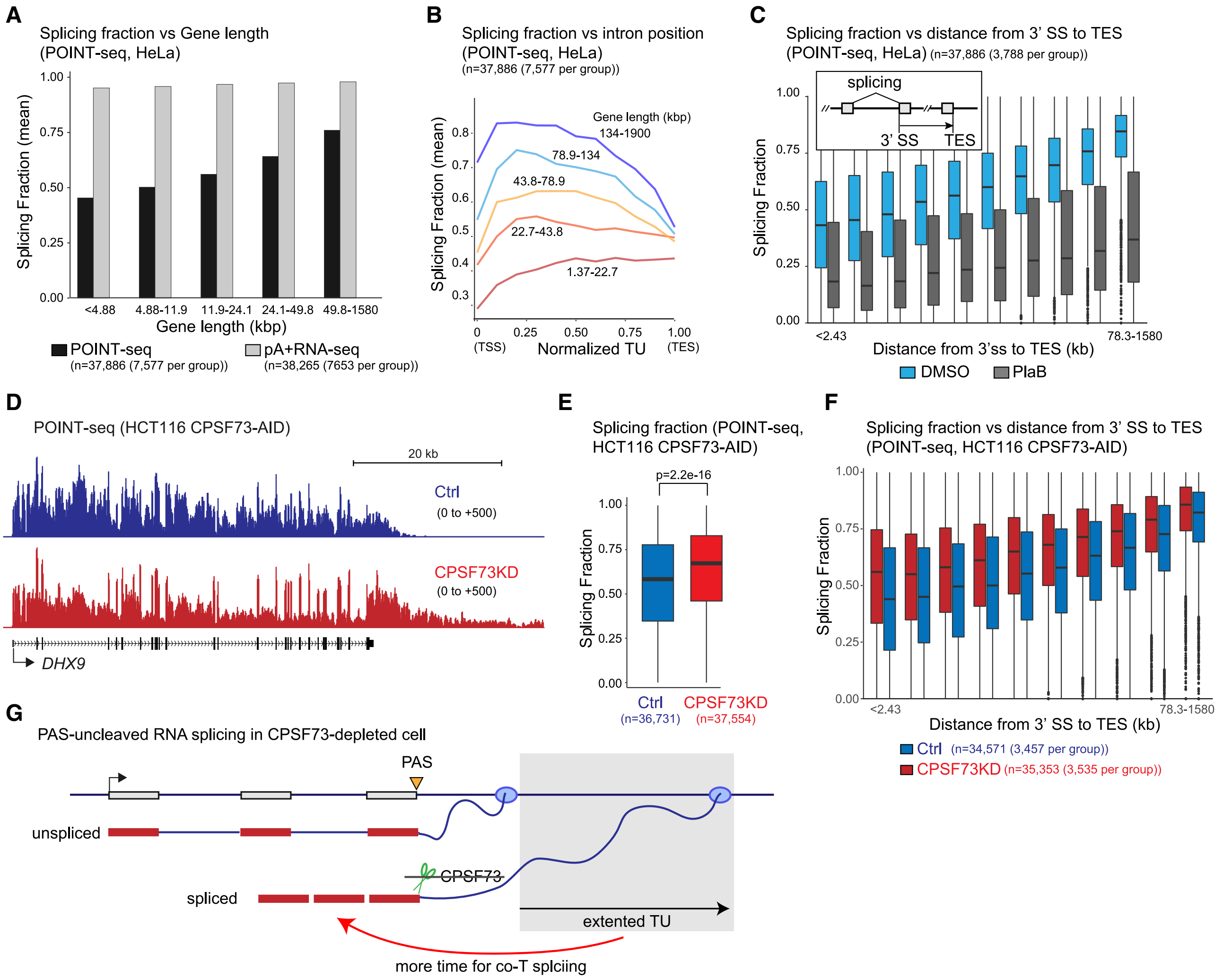

Figure 7. Co-T splicing following RNA cleavage at PAS

(A) Splicing fraction of POINT-seq (black) and pA+ RNA-seq (gray) from HeLa cells with different gene lengths.

(B) Splicing fraction in each intron position of normalized TU with five different gene lengths as indicated. POINT-seq analysis in HeLa cells.

(C) Splicing fraction of POINT-seq from DMSO-treated (light blue) or PlaB-treated (dark gray) HeLa cells with indicated distance from 3′ SS to TES.

(D) POINT-seq profiles of *DHX9* in CPSF73KD (red) and Ctrl (blue) HCT116 cells.

(E) Splicing fraction of POINT-seq signals in CPSF73KD (red) and Ctrl (blue) HCT116 cells.

(F) Splicing fraction of POINT-seq signals in CPSF73KD (red) and Ctrl (blue) HCT116 cells with indicated distance from 3′ SS to TES.

(G) Model of co-T splicing regulated by defects of RNA cleavage and transcription termination. Orange triangle is PAS. Green scissor shows RNA cleavage activity of CPSF73. Red arrow denotes enhancement of co-T splicing.

were similar for both short and long exons, even though the distance from the 3′ SS at which spliced reads decline was predictably shorter for the shortest exons (Figure S7A). Similar levels of spliced reads were detected on the downstream intron irrespective of exon size (Figure S7B). In contrast, longer introns were associated with a higher proportion of spliced reads corresponding to both immediate (Figure S7C) and delayed splicing (Figures S7D and S7E). As long introns are generally present in long genes, likely long genes are more efficiently spliced than short genes. POINT-seq datasets confirm that indeed the proportion of spliced reads increases with gene length. As a control, we calculated the proportion of spliced reads detected in libraries prepared from pA+ RNA fraction, which gave largely spliced reads irrespective of gene length (Figure 7A).

We next analyzed the proportion of spliced reads associated with introns located at different positions along the TU (Figure 7B). The splicing fraction of the first intron was generally lower than internal introns, as previously reported in *S. pombe* (Herzel et al., 2018). Moreover, the splicing fraction gradually decreased from the middle of the TU toward the TES, except for the shortest gene class (Figure 7B). This suggests that splicing levels depend on the time that Pol II spends elongating past the 3′ SS. Indeed, analysis of POINT-seq datasets shows that the proportion of spliced reads increased with the distance

from the 3′ SS to TES (Figure 7C). PlaB treatment confirmed the authenticity of spliced reads detected by POINT-seq. We also analyzed the effect on splicing of the distance to TES from 3′ SSs located at different positions along a normalized TU. This shows similar effects for all positions with both constitutive and alternatively spliced exons (Figures S7F and S7G).

Our bioinformatic analysis of splicing shows a clear kinetic effect whereby longer introns or longer genes allow more time to complete successful splicing. We therefore reasoned that inducing an artificial extension to Pol II TUs could also act to enhance splicing levels. This was tested by CPSF73-AID-induced degradation for 3 h (Figure S3H), followed by POINT-seq analysis, confirming that read-through transcription occurs at gene ends, as shown for *DHX9* (Figure 7D). We further observed that upon CPSF73 depletion, transcription termination defects at the end of *HNRNPA0* caused transcript read-in on normally silent *KLHL3*. Similar effects were observed for two other PC genes, *PRPF38B* and *BROX* (Figure S7H). Interestingly spliced reads corresponding to these transcripts were in all cases detected, indicating that Pol II termination defects do not inhibit splicing. Consistently, herpes simplex virus (HSV) infection and SETD2 mutation in clear cell renal cell carcinoma, which disrupt transcriptional termination, both generate extended host gene transcripts between adjacent genes that are often spliced between normally separate TUs (Grosso et al., 2015; Rutkowski et al., 2015).

We finally focused on the proportion of spliced reads along PC genes and found a significant increase in CPSF73-depleted cells (Figures 7E and S7I). Enhanced splicing levels were observed, as shown by a relative enrichment of exonic signals in *DHX9* transcripts (Figure 7D) and by quantitative analysis of the proportion of spliced reads at different distances from the TES (Figure 7F). Overall, our analysis of extended TUs caused by CPSF73 depletion reveals that upstream splicing efficiency is enhanced, presumably by providing more time for assembly of a catalytically active spliceosome (Figure 7G).

DISCUSSION

POINT methodology

Several sequencing technologies have been developed to profile mammalian nascent transcription (Stark et al., 2019). GRO-seq and PRO-seq use *in vitro* nuclear run-on (NRO) transcription with modified ribonucleotides to label and isolate newly synthesized RNA (Core and Lis, 2008; Kwak et al., 2013). Metabolic labeling of nascent RNA can also be carried out on cell cultures by application of 4sU-seq and TT-seq (Schwalb et al., 2016; Windhager et al., 2012). These methods allow analysis of Pol II pausing, elongation speed, and RNA stability. Furthermore, sequencing chromatin-associated RNA 3′ ends can be used as a surrogate to sequence Pol II active site-associated transcripts (Mayer et al., 2015). However, it is apparent that chromatin-associated RNA contains significant amounts of contaminating steady-state mRNA (Schlackow et al., 2017). We previously developed mammalian mNET-seq (Nojima et al., 2015) to circumvent nucleoplasmic contamination issues. In mNET-seq, both accessible DNA and RNA in the chromatin fraction are digested by MNase treatment, and the transcription machinery is then immunoprecipitated with Pol II antibody from the solubilized chromatin. Sequencing the protected short RNA fragment within the Pol II complex effectively defines the nascent RNA 3′ end. mNET-seq has allowed the characterization of Pol II pausing and the detection of splicing intermediates (Nojima et al., 2015, 2018a). However, the restricted read length obtained by mNET-seq has precluded the analysis of RNA processing kinetics in mammalian cells. Here we describe POINT technology, which provides a new method to profile intact and truly nascent RNA. Notably, a rRNA depletion step routinely used in most RNA-seq methodologies is unnecessary for POINT technology. This is because a high concentration of strong detergent (3% Empigen) is added during both chromatin isolation and Pol II IP steps, which removes all contaminating RNA species without affecting Pol II IP. These contaminating RNAs, such as mature spliced transcripts are often detected in other nascent RNA labeling approaches (Barbieri et al., 2020). Consequently, POINT technology enables the rapid purification of a truly nascent RNA preparation. As a way to emphasize the utility of our POINT technology, we have directly used POINT-5, POINT-seq, and POINT-nano to better understand how co-T RNA processing of nascent transcription by Pol II is executed.

POINT-5 analysis reveals different categories of transcript cleavage

POINT-5 provides powerful methodology to map transcript 5′ ends that rivals the widely used CAGE technique (Figures 1E and 1F). CAGE has been applied to map TSS of steady-state RNA and in some cases chromatin-bound RNA (Hirabayashi et al., 2019). Notably, our POINT-5 method not only defines TSS positions but also distinguishes co-T RNA cleavage sites corresponding to 5′ P-RNA from TSS by use of the 5′ P-specific exonuclease, ExT. We show that RNA cleavage at the PAS is readily detected by POINT-5 only following Xrn2 depletion. This confirms that the downstream product of PAS cleavage is rapidly degraded by Xrn2 to promote Pol II transcriptional termination (Proudfoot, 2016). Instead, POINT-5 analysis detected 3′ end cleavage of pri-miRNA, pre-U snRNA, and pre-histone transcripts without prior Xrn2 depletion. For these gene classes, it is known that RNA cleavage is required to induce their transcription termination. However, Xrn2 loss did not significantly affect transcription termination of U snRNA and histone genes in POINT-seq (Figure S3E), as previously shown by mNET-seq analysis (Eaton et al., 2018). Either these cleaved RNAs are not subject to degradation or possibly are degraded by other 5′-3′ exonucleases such as Xrn1, which although mainly localized in the cytoplasm may also possess nuclear functions (Sohrabi-Jahromi et al., 2019).

PTT is a critically important step to fine-tune gene expression (Kamieniarz-Gdula and Proudfoot, 2019). Previously, U1 snRNP was shown to suppress cleavage at intronic cryptic PAS and thereby restrict PTT by telescripting (Kaida et al., 2010). However, we find that a significant number of genes with PTT suppressed by U1 snRNP are also regulated by PlaB-sensitive SF3B1, a component of U2 snRNP (Figure S4A). This suggests that splicing per se is often directly connected to PTT. Additionally, such PTT induced by PlaB is significantly extended by CPSF73 depletion (55% of gene cases) and Xrn2 depletion

(65% of gene cases), suggesting that in these cases PTT is PAS dependent. In contrast, we also observe Xrn2-independent POINT-5 peaks, sensitive to ExT treatment near the TSS of PC genes (Figure S3G). Possibly the Integrator complex is responsible for these cleavage sites as a way to regulate PTT. Indeed, recent studies have shown that Integrator complex prematurely terminates PC genes (Elrod et al., 2019; Tatomer et al., 2019) and also terminates transcription of lncRNA genes (Lai et al., 2015). This suggests that PTT may be regulated by a combination of CPA and Integrator complexes, similar to termination of lncRNA transcription (Nojima et al., 2018b).

POINT-nano defines different kinetic classes of co-T splicing

On the basis of POINT-nano analysis, we observe two main categories of co-T splicing: immediate and delayed. First, splicing can occur to a 40% level immediately following intron synthesis, with elongating Pol II still within the downstream exon. We note that abundant immediate splicing has also been recently reported in murine erythroleukemic (MEL) cells for PC gene introns (Reimer et al., 2021). Instead, if elongating Pol II reaches the following intron, then splicing levels are initially lower but gradually increase as Pol II elongates through the intron and further into 3′ regions of the TU. Notably high-resolution chromatin immunoprecipitation sequencing (ChIP-seq) and mNET-seq data reveal that Pol II CTD S5P is enriched over exons where it acts to recruit active spliceosomes. Subsequently, CTD phosphorylation status changes to S2P as Pol II elongates into downstream introns (Chathoth et al., 2014; Nojima et al., 2018a). Such a CTD phosphorylation transition may be important to control Pol II elongation speed during transcription. Indeed, our current model is that Pol II is paused over exons to allow immediate splicing but then speeds up following completion of splicing, likely because of the release of the spliceosome from the elongation complex. Nucleosome positioning may also contribute to the regulation of immediate splicing by slowing down Pol II at the intron-exon boundary (Schwartz et al., 2009; Tilgner et al., 2009). We note that a related study using ONT did not detect immediate splicing in human cells (Drexler et al., 2020). However, their use of 4sU labeling to isolate nascent RNAs could potentially cause a bias with U-rich intronic RNA. Also, their chromatin-derived RNA may be significantly contaminated with rRNA and fully spliced mature mRNA and so may underscore the levels of co-T splicing.

Another notable feature of our splicing analysis by POINT-nano is that splicing increases to higher levels with longer TUs. Thus, either greater intron or gene size correlates with increased levels of splicing. These correlations imply a simple kinetic model whereby more Pol II elongation time allows more productive spliceosome assembly or splicing completion on upstream introns. However, we also note that a fraction of transcripts appears refractory to both splicing and PAS-mediated 3′ end processing. A similar correlation between inefficient splicing and lack of 3′ end processing has also been made for highly expressed globin genes in MEL cells (Reimer et al., 2021). We predict that such transcripts may be defective and likely subjected to degradation by nuclear quality control. As a way to test the hypothesis that longer TUs correlate with higher splicing efficiency, we tested the effect of abrogating PAS cleavage by CPSF73 depletion. These data confirmed that extending TU length by preventing Pol II termination did indeed promote more efficient splicing of upstream introns. This albeit artificial situation may have relevance to the regulation of gene expression though alternative PAS selection. In particular a switch from proximal to distal PAS that is correlated with cellular differentiation (Di Giammartino et al., 2011) may be able to stimulate upstream splicing events.

Limitations of study

POINT-nano

This uses a direct cDNA sequencing approach by ONT with no prior PCR amplification. Consequently, we used higher RNA input for POINT-nano (>500 ng) than POINT-seq (50 ng), as only the later Illumina-based approach uses amplification. POINT-nano analysis of single cells or small cell number samples is currently not possible. Even with larger cell numbers (as from cell culture), gene read coverage of the human genome was limited in our POINT-nano analysis. Consequently, it was necessary to focus on more highly expressed genes to study complete TUs. Additionally, we currently have a read length limitation to our POINT-nano data. Although input RNA was on average 6,000 nt or greater (Figure S5A), mean read length was restricted about 1,200 nt (Figure 5C). Consequently, the RNA 5′ ends derived from our direct cDNA sequencing will be a mix of authentic 5′ termini and artificially truncated reads, as 5′ ends of POINT-nano were not always consistent with POINT-5 peaks in middle of the gene (see *ACTB* profile in Figure 5B). Obtaining longer POINT-nano reads that match the RNA input will be invaluable to obtain a more complete profile of PC gene TUs.

POINT-seq

Another feature of this technique is that 5′ ends of TUs will be under-represented because of removal of the terminal ~150 nt during Illumina total RNA sequencing library preparation. This will lead to reduced TSS-associated sequence and will therefore underscore the high levels of TSS-associated PTT/pausing observed for most PC genes, which can be detected using the mNET-seq method (Nojima et al., 2015).

Despite these limitations, it is clear that our POINT technology illuminates a deeper understanding of PC gene nascent transcription and coupled RNA processing.

STAR★METHODS

Detailed methods are provided in the online version of this paper and include the following:

- KEY RESOURCES TABLE
- RESOURCE AVAILABILITY
  - Lead contact
  - Materials availability
  - Data and code availability
- EXPERIMENTAL MODEL AND SUBJECT DETAILS
- METHOD DETAILS
  - Auxin-dependent protein depletion
  - POINT methodology and library prep
  - POINT-seq
  - POINT-5

- ○ POINT-nano
- ○ Sequencing service
- ○ Exoterminator treatment
- QUANTIFICATION AND STATISTICAL ANALYSIS
  - ○ Illumina data pre-processing
  - ○ POINT-nano pre-processing
  - ○ Identification of expressed genes
  - ○ Metagene Analysis
  - ○ Heatmaps
  - ○ Cleavage ratio
  - ○ Termination index
  - ○ Premature transcription termination (PTT) analysis
  - ○ Splicing analyses with POINT technology
  - ○ Alternative splicing events and cassette cases identification
  - ○ Signal extraction and data visualization
  - ○ Reads quantification
  - ○ P values and significance tests

SUPPLEMENTAL INFORMATION

Supplemental information can be found online at https://doi.org/10.1016/j.molcel.2021.02.034.

ACKNOWLEDGMENTS

We thank members of the N.J.P. and M.C.-F. groups, especially Rosina Savisaar, for critical discussion. We also thank Steven West for providing us HCT116 Xrn2-AID and CPSF73-AID cells and critical comments on this paper. This work was supported by funding to N.J.P. (Wellcome Trust Investigator Award [107928/Z/15/Z] and European Research Council [ERC] Advanced [339270] Grants) and to M.C.-F. and R.S.-L. (Fundação para Ciência Tecnologia, Portugal [PTDC/MED-ONC/29469/2017] and Fellowship SFRH/BD/147906/2019). Funding was also received from the European Union (EU) Horizon 2020 Research and Innovation Programme (RiboMed 857119).

AUTHOR CONTRIBUTIONS

R.S.-L. conducted the bioinformatic analyses. T.N. performed the molecular biology experiments with help from G.D. for biochemical experiments and interpretation of splicing data and from I.Z. for Xrn2 protein analysis. H.K. provided valuable Pol II antibodies. C.W. provided insight into use of ONT. R.S.-L., M.C.-F., N.J.P., and T.N. designed the project and co-wrote the paper.

DECLARATION OF INTERESTS

The authors declare no competing interests.

Received: November 3, 2020
Revised: December 21, 2020
Accepted: February 24, 2021
Published: March 17, 2021

REFERENCES

Andersson, R., and Sandelin, A. (2020). Determinants of enhancer and promoter activities of regulatory elements. Nat. Rev. Genet. *21*, 71–87.

Barbieri, E., Hill, C., Quesnel-Vallières, M., Zucco, A.J., Barash, Y., and Gardini, A. (2020). Rapid and scalable profiling of nascent RNA with fastGRO. Cell Rep. *33*, 108373.

Bonnet, A., Grosso, A.R., Elkaoutari, A., Coleno, E., Presle, A., Sridhara, S.C., Janbon, G., Géli, V., de Almeida, S.F., and Palancade, B. (2017). Introns protect eukaryotic genomes from transcription-associated genetic instability. Mol. Cell *67*, 608–621.e6.

Bray, N.L., Pimentel, H., Melsted, P., and Pachter, L. (2016). Near-optimal probabilistic RNA-seq quantification. Nat. Biotechnol. *34*, 525–527.

Chan, P.P., and Lowe, T.M. (2016). GtRNAdb 2.0: an expanded database of transfer RNA genes identified in complete and draft genomes. Nucleic Acids Res. *44* (D1), D184–D189.

Chathoth, K.T., Barrass, J.D., Webb, S., and Beggs, J.D. (2014). A splicing-dependent transcriptional checkpoint associated with prespliceosome formation. Mol. Cell *53*, 779–790.

Chen, J., and Wagner, E.J. (2010). snRNA 3′ end formation: the dawn of the Integrator complex. Biochem. Soc. Trans. *38*, 1082–1087.

Chen, Y., Pai, A.A., Herudek, J., Lubas, M., Meola, N., Järvelin, A.I., Andersson, R., Pelechano, V., Steinmetz, L.M., Jensen, T.H., and Sandelin, A. (2016). Principles for RNA metabolism and alternative transcription initiation within closely spaced promoters. Nat. Genet. *48*, 984–994.

Core, L.J., and Lis, J.T. (2008). Transcription regulation through promoter-proximal pausing of RNA polymerase II. Science *319*, 1791–1792.

De Coster, W., D'Hert, S., Schultz, D.T., Cruts, M., and Van Broeckhoven, C. (2018). NanoPack: visualizing and processing long-read sequencing data. Bioinformatics *34*, 2666–2669.

Dhir, A., Dhir, S., Proudfoot, N.J., and Jopling, C.L. (2015). Microprocessor mediates transcriptional termination of long noncoding RNA transcripts hosting microRNAs. Nat. Struct. Mol. Biol. *22*, 319–327.

Di Giammartino, D.C., Nishida, K., and Manley, J.L. (2011). Mechanisms and consequences of alternative polyadenylation. Mol. Cell *43*, 853–866.

Dobin, A., Davis, C.A., Schlesinger, F., Drenkow, J., Zaleski, C., Jha, S., Batut, P., Chaisson, M., and Gingeras, T.R. (2013). STAR: ultrafast universal RNA-seq aligner. Bioinformatics *29*, 15–21.

Drexler, H.L., Choquet, K., and Churchman, L.S. (2020). Splicing kinetics and coordination revealed by direct nascent RNA sequencing through nanopores. Mol. Cell *77*, 985–998.e8.

Eaton, J.D., Davidson, L., Bauer, D.L.V., Natsume, T., Kanemaki, M.T., and West, S. (2018). Xrn2 accelerates termination by RNA polymerase II, which is underpinned by CPSF73 activity. Genes Dev. *32*, 127–139.

Eaton, J.D., Francis, L., Davidson, L., and West, S. (2020). A unified allosteric/torpedo mechanism for transcriptional termination on human protein-coding genes. Genes Dev. *34*, 132–145.

Elrod, N.D., Henriques, T., Huang, K.L., Tatomer, D.C., Wilusz, J.E., Wagner, E.J., and Adelman, K. (2019). The Integrator complex attenuates promoter-proximal transcription at protein-coding genes. Mol. Cell *76*, 738–752.e7.

Grosso, A.R., Leite, A.P., Carvalho, S., Matos, M.R., Martins, F.B., Vítor, A.C., Desterro, J.M., Carmo-Fonseca, M., and de Almeida, S.F. (2015). Pervasive transcription read-through promotes aberrant expression of oncogenes and RNA chimeras in renal carcinoma. eLife *4*, e09214.

Ha, M., and Kim, V.N. (2014). Regulation of microRNA biogenesis. Nat. Rev. Mol. Cell Biol. *15*, 509–524.

Herzel, L., Straube, K., and Neugebauer, K.M. (2018). Long-read sequencing of nascent RNA reveals coupling among RNA processing events. Genome Res. *28*, 1008–1019.

Hirabayashi, S., Bhagat, S., Matsuki, Y., Takegami, Y., Uehata, T., Kanemaru, A., Itoh, M., Shirakawa, K., Takaori-Kondo, A., Takeuchi, O., et al. (2019). NET-CAGE characterizes the dynamics and topology of human transcribed cis-regulatory elements. Nat. Genet. *51*, 1369–1379.

Itoh, H., Washio, T., and Tomita, M. (2004). Computational comparative analyses of alternative splicing regulation using full-length cDNA of various eukaryotes. RNA *10*, 1005–1018.

Kaida, D., Berg, M.G., Younis, I., Kasim, M., Singh, L.N., Wan, L., and Dreyfuss, G. (2010). U1 snRNP protects pre-mRNAs from premature cleavage and polyadenylation. Nature *468*, 664–668.

Kamieniarz-Gdula, K., and Proudfoot, N.J. (2019). Transcriptional control by premature termination: a forgotten mechanism. Trends Genet. *35*, 553–564.

Kent, W.J., Sugnet, C.W., Furey, T.S., Roskin, K.M., Pringle, T.H., Zahler, A.M., and Haussler, D. (2002). The Human Genome Browser at UCSC. Genome Res. *12*, 996–1006.

Kotake, Y., Sagane, K., Owa, T., Mimori-Kiyosue, Y., Shimizu, H., Uesugi, M., Ishihama, Y., Iwata, M., and Mizui, Y. (2007). Splicing factor SF3b as a target of the antitumor natural product pladienolide. Nat. Chem. Biol. *3*, 570–575.

Kwak, H., Fuda, N.J., Core, L.J., and Lis, J.T. (2013). Precise maps of RNA polymerase reveal how promoters direct initiation and pausing. Science *339*, 950–953.

Lai, F., Gardini, A., Zhang, A., and Shiekhattar, R. (2015). Integrator mediates the biogenesis of enhancer RNAs. Nature *525*, 399–403.

Li, H. (2018). Minimap2: pairwise alignment for nucleotide sequences. Bioinformatics *34*, 3094–3100.

Li, H., Handsaker, B., Wysoker, A., Fennell, T., Ruan, J., Homer, N., Marth, G., Abecasis, G., and Durbin, R.; 1000 Genome Project Data Processing Subgroup (2009). The Sequence Alignment/Map format and SAMtools. Bioinformatics *25*, 2078–2079.

Mandel, C.R., Kaneko, S., Zhang, H., Gebauer, D., Vethantham, V., Manley, J.L., and Tong, L. (2006). Polyadenylation factor CPSF-73 is the pre-mRNA 3′-end-processing endonuclease. Nature *444*, 953–956.

Martinez-Rucobo, F.W., Kohler, R., van de Waterbeemd, M., Heck, A.J., Hemann, M., Herzog, F., Stark, H., and Cramer, P. (2015). Molecular basis of transcription-coupled pre-mRNA capping. Mol. Cell *58*, 1079–1089.

Marzluff, W.F., and Koreski, K.P. (2017). Birth and death of histone mRNAs. Trends Genet. *33*, 745–759.

Mayer, A., di Iulio, J., Maleri, S., Eser, U., Vierstra, J., Reynolds, A., Sandstrom, R., Stamatoyannopoulos, J.A., and Churchman, L.S. (2015). Native elongating transcript sequencing reveals human transcriptional activity at nucleotide resolution. Cell *161*, 541–554.

Morlando, M., Ballarino, M., Gromak, N., Pagano, F., Bozzoni, I., and Proudfoot, N.J. (2008). Primary microRNA transcripts are processed co-transcriptionally. Nat. Struct. Mol. Biol. *15*, 902–909.

Nojima, T., Gomes, T., Grosso, A.R.F., Kimura, H., Dye, M.J., Dhir, S., Carmo-Fonseca, M., and Proudfoot, N.J. (2015). Mammalian NET-seq reveals genome-wide nascent transcription coupled to RNA processing. Cell *161*, 526–540.

Nojima, T., Gomes, T., Carmo-Fonseca, M., and Proudfoot, N.J. (2016). Mammalian NET-seq analysis defines nascent RNA profiles and associated RNA processing genome-wide. Nat. Protoc. *11*, 413–428.

Nojima, T., Rebelo, K., Gomes, T., Grosso, A.R., Proudfoot, N.J., and Carmo-Fonseca, M. (2018a). RNA polymerase II phosphorylated on CTD serine 5 interacts with the spliceosome during co-transcriptional splicing. Mol. Cell *72*, 369–379.e4.

Nojima, T., Tellier, M., Foxwell, J., Ribeiro de Almeida, C., Tan-Wong, S.M., Dhir, S., Dujardin, G., Dhir, A., Murphy, S., and Proudfoot, N.J. (2018b). Deregulated expression of mammalian lncRNA through loss of SPT6 induces R-loop formation, replication stress, and cellular senescence. Mol. Cell *72*, 970–984.e7.

Oesterreich, F.C., Herzel, L., Straube, K., Hujer, K., Howard, J., and Neugebauer, K.M. (2016). Splicing of nascent RNA coincides with intron exit from RNA polymerase II. Cell *165*, 372–381.

Oh, J.M., Di, C., Venters, C.C., Guo, J., Arai, C., So, B.R., Pinto, A.M., Zhang, Z., Wan, L., Younis, I., and Dreyfuss, G. (2017). U1 snRNP telescripting regulates a size-function-stratified human genome. Nat. Struct. Mol. Biol. *24*, 993–999.

Pawlicki, J.M., and Steitz, J.A. (2008). Primary microRNA transcript retention at sites of transcription leads to enhanced microRNA production. J. Cell Biol. *182*, 61–76.

Preker, P., Nielsen, J., Kammler, S., Lykke-Andersen, S., Christensen, M.S., Mapendano, C.K., Schierup, M.H., and Jensen, T.H. (2008). RNA exosome depletion reveals transcription upstream of active human promoters. Science *322*, 1851–1854.

Proudfoot, N.J. (2016). Transcriptional termination in mammals: Stopping the RNA polymerase II juggernaut. Science *352*, aad9926.

Quinlan, A.R., and Hall, I.M. (2010). BEDTools: a flexible suite of utilities for comparing genomic features. Bioinformatics *26*, 841–842.

Ramírez, F., Dündar, F., Diehl, S., Grüning, B.A., and Manke, T. (2014). deepTools: a flexible platform for exploring deep-sequencing data. Nucleic Acids Res. *42*, W187–W191.

Ramírez, F., Ryan, D.P., Grüning, B., Bhardwaj, V., Kilpert, F., Richter, A.S., Heyne, S., Dündar, F., and Manke, T. (2016). deepTools2: a next generation web server for deep-sequencing data analysis. Nucleic Acids Res. *44* (W1), W160–W165.

Reimer, K.A., Mimoso, C., Adelman, K., and Neugebauer, K.M. (2021). Co-transcriptional splicing regulates 3' end cleavage during mammalian erythropoiesis. Molecular cell. https://doi.org/10.1016/j.molcel.2020.12.018.

Rutkowski, A.J., Erhard, F., L'Hernault, A., Bonfert, T., Schilhabel, M., Crump, C., Rosenstiel, P., Efstathiou, S., Zimmer, R., Friedel, C.C., and Dölken, L. (2015). Widespread disruption of host transcription termination in HSV-1 infection. Nat. Commun. *6*, 7126.

Schlackow, M., Nojima, T., Gomes, T., Dhir, A., Carmo-Fonseca, M., and Proudfoot, N.J. (2017). Distinctive patterns of transcription and RNA processing for human lincRNAs. Mol. Cell *65*, 25–38.

Schwalb, B., Michel, M., Zacher, B., Frühauf, K., Demel, C., Tresch, A., Gagneur, J., and Cramer, P. (2016). TT-seq maps the human transient transcriptome. Science *352*, 1225–1228.

Schwartz, S., Meshorer, E., and Ast, G. (2009). Chromatin organization marks exon-intron structure. Nat. Struct. Mol. Biol. *16*, 990–995.

So, B.R., Di, C., Cai, Z., Venters, C.C., Guo, J., Oh, J.M., Arai, C., and Dreyfuss, G. (2019). A complex of U1 snRNP with cleavage and polyadenylation factors controls telescripting, regulating mRNA transcription in human cells. Mol. Cell *76*, 590–599.e4.

Sohrabi-Jahromi, S., Hofmann, K.B., Boltendahl, A., Roth, C., Gressel, S., Baejen, C., Soeding, J., and Cramer, P. (2019). Transcriptome maps of general eukaryotic RNA degradation factors. eLife *8*, e47040.

Stark, R., Grzelak, M., and Hadfield, J. (2019). RNA sequencing: the teenage years. Nat. Rev. Genet. *20*, 631–656.

Tapial, J., Ha, K.C.H., Sterne-Weiler, T., Gohr, A., Braunschweig, U., Hermoso-Pulido, A., Quesnel-Vallières, M., Permanyer, J., Sodaei, R., Marquez, Y., et al. (2017). An atlas of alternative splicing profiles and functional associations reveals new regulatory programs and genes that simultaneously express multiple major isoforms. Genome Res. *27*, 1759–1768.

Tatomer, D.C., Elrod, N.D., Liang, D., Xiao, M.S., Jiang, J.Z., Jonathan, M., Huang, K.L., Wagner, E.J., Cherry, S., and Wilusz, J.E. (2019). The Integrator complex cleaves nascent mRNAs to attenuate transcription. Genes Dev. *33*, 1525–1538.

Tilgner, H., Nikolaou, C., Althammer, S., Sammeth, M., Beato, M., Valcárcel, J., and Guigó, R. (2009). Nucleosome positioning as a determinant of exon recognition. Nat. Struct. Mol. Biol. *16*, 996–1001.

Uguen, P., and Murphy, S. (2003). The 3′ ends of human pre-snRNAs are produced by RNA polymerase II CTD-dependent RNA processing. EMBO J. *22*, 4544–4554.

Ule, J., and Blencowe, B.J. (2019). Alternative splicing regulatory networks: functions, mechanisms, and evolution. Mol. Cell *76*, 329–345.

West, S., Gromak, N., and Proudfoot, N.J. (2004). Human 5′–> 3′ exonuclease Xrn2 promotes transcription termination at co-transcriptional cleavage sites. Nature *432*, 522–525.

Windhager, L., Bonfert, T., Burger, K., Ruzsics, Z., Krebs, S., Kaufmann, S., Malterer, G., L'Hernault, A., Schilhabel, M., Schreiber, S., et al. (2012). Ultrashort and progressive 4sU-tagging reveals key characteristics of RNA processing at nucleotide resolution. Genome Res. *22*, 2031–2042.

Yeo, G., and Burge, C.B. (2004). Maximum entropy modeling of short sequence motifs with applications to RNA splicing signals. J. Comput. Biol. *11*, 377–394.

CellPress
OPEN ACCESS

STAR★METHODS

KEY RESOURCES TABLE

REAGENT OR RESOURCE	SOURCE	IDENTIFIER
**Reagent**		
Auxin (IAA)	Sigma	Cat# I2886
Tetracycline (Tet)	Sigma	Cat# 87128
Empigen ~30%	Sigma	Cat# 30326
Turbo DNase	Thermo Fisher Scientific	Cat# AM2239
RiboLock RNase inhibitor	Thermo Fisher Scientific	Cat# EO0381
Dynabeads M280 Sheep Anti-mouse IgG	Thermo Fisher Scientific	Cat# 11202D
E. coli Poly A polymerase	NEB	Cat# M0276
Pladienolide B	Santa Cruz	Cat# SC-391691
DRB	Sigma	Cat#D1916
Terminator 5-Phosphate-Dependent Exonuclease (Exoterminator)	Cambio	Cat#TER51020
SPRISelect reagent	Beckman Coulter	Cat# B23317
Spike-in SIRV-Set2	Lexogen	Cat#050.0
**Antibodies**		
Mouse monoclonal anti-Pol II CTD, Total	Nojima et al., 2015	CMA601, Available from Kimura Lab by request.
Mouse monoclonal anti-H3	Active Motif	Cat# 39763; RRID:AB_2650522
Xrn2	Bethyl Laboratories	Cat# A301-101A; RRID:AB_873178
Tubulin	Abcam	Cat# ab7291; RRID:AB_2241126
mAID	MBL	Cat#M214-3; RRID:AB_2890014
**Deposited data**		
Raw sequencing data	This study	GEO: GSE159326
Re-analyzed ChrRNA-seq data	Nojima et al., 2015	GEO: GSE60358
Re-analyzed pA+ RNA-seq HeLa S3 data	https://www.genome.gov/27528022	GEO: GSE86661
Re-analyzed pA+ RNA-seq HCT116 data	https://www.genome.gov/27528022	GEO: GSE33480
Re-analyzed U1 AMO 4shU-seq data	Oh et al., 2017	GEO: GSE103252
Re-analyzed CAGE data	Chen et al., 2016	GEO: GSE75183
Re-analyzed mNET-seq	Schlackow et al., 2017	GEO: GSE81662
**Cell lines**		
HeLa (human)	Proudfoot Lab	N/A
Xrn2-AID HCT116 (human)	Eaton et al., 2018	Available from West Lab by request.
CPSF73-AID HCT116 (human)	Eaton et al., 2020	Available from West Lab by request.
**Gels**		
Novex 6% TBE gel, 12 well	Invitrogen	Cat# EC62652BOX
4-15% Mini-PROTEAN Precast Protein Gels, 12 well	BioRad	Cat# 4561085
**Kits**		
NEBNext Ultra II Directional RNA library prep kit for Illumina	NEB	Cat# E7760S (Note: this kit for POINT-seq)
SMARTer Stranded RNA-seq kit for Illumina	Takara Bio	Cat# 634836 (Note: this kit for POINT-5)
Direct cDNA library prep kit for ONT	Oxford Nanopore Technologies (ONT)	Cat#SQK-DCS109 (Note: this kit for POINT-nano)
PromethION48 flow cell	Oxford Nanopore Technologies (ONT)	Cat# FLO-POR002

(*Continued on next page*)

Continued		
REAGENT OR RESOURCE	SOURCE	IDENTIFIER
Nanopore barcoding kit	Oxford Nanopore Technologies (ONT)	Cat# EXP-NBD114
Direct-zol RNA microprep	Zymo Research	Cat# R2061
High Sensitivity RNA ScreenTape for TapeStation	Agilent	Car# 5067-5579
High Sensitivity RNA ScreenTape Sample Buffer for TapeStation	Agilent	Car# 5067-5580
High Sensitivity RNA ScreenTape Ladder for TapeStation	Agilent	Cat# 5067-5581
Software and algorithms		
FastQC (v0.11.5)	https://www.bioinformatics.babraham.ac.uk/projects/fastqc/	N/A
TrimGalore (v0.4.4)	https://www.bioinformatics.babraham.ac.uk/projects/trim_galore/	N/A
STAR (v2.7.0)	https://github.com/alexdobin/STAR	Dobin et al., 2013
SAMtools (v1.9)	http://www.htslib.org/	Li et al., 2009
BedTools (v2.29.2)	https://bedtools.readthedocs.io/en/latest/content/installation.html	Quinlan and Hall, 2010
Deeptools (v3.4.3)	https://deeptools.readthedocs.io/en/latest/index.html	Ramírez et al., 2016
Guppy Basecalling (v3.0.5)	Oxford Nanopore Technologies (ONT)	N/A
NanoQC (v0.9.1)	https://github.com/wdecoster/nanoQC	De Coster et al., 2018
qcat (v1.1.0)	https://github.com/nanoporetech/qcat	N/A
regEx (v2.5.76)	https://pypi.org/project/regex/	N/A
Porechop (v0.2.4)	https://github.com/rrwick/Porechop	N/A
minimap2 (v2.17-r941)	https://github.com/lh3/minimap2	Li, 2018
pysam (v0.15.4)	https://github.com/pysam-developers/pysam	Li et al., 2009
Kallisto (v0.46.0)	https://github.com/pachterlab/kallisto	Bray et al., 2016
ggplot (v3.3.2)	https://cran.r-project.org/web/packages/ggplot2/index.html, https://ggplot2.tidyverse.org.	N/A
MaxEntScan	http://hollywood.mit.edu/burgelab/maxent/Xmaxentscan_scoreseq.html	Yeo and Burge, 2004
scales (v1.1.1)	https://scales.r-lib.org/	N/A
vast-tools (v2.5.1)	https://github.com/vastgroup/vast-tools	Tapial et al., 2017
bedGraphToBigWig (v4)	https://www.encodeproject.org/software/bedgraphtobigwig/	N/A

RESOURCE AVAILABILITY

Lead contact

Further information and requests for resources and reagents should be directed to Takayuki Nojima (taka.nojima@path.ox.ac.uk or taka.nojima@bioreg.kyushu-u.ac.jp).

Materials availability

All reagents in this study are commercially available as indicated in Key Resources Table except for anti-Pol II CTD antibody which was provided by Dr. Hiroshi Kimura.

Data and code availability

The raw and processed data derived from POINT-seq, POINT-5 and POINT-nano analyses as generated in this study are deposited in NCBI GEO (GSE159326). The associated raw image data are available from Mendeley (https://doi.org/10.17632/wsgjzvzs26.1). All code supporting POINT analyses are available on request. The reanalysed published data used in this study can be found at GEO as indicated in the Key Resources Table.

EXPERIMENTAL MODEL AND SUBJECT DETAILS

HeLa and HCT116 cells were maintained in high glucose Dulbecco's Modified Eagle's Medium (DMEM) with 10% fetal bovine serum (FBS) and penicillin/streptomycin (PS) at 37°C with 5% CO_2.

METHOD DETAILS

Auxin-dependent protein depletion

IAA (final concentration 0.5 mM) was directly added to Xrn2-AID HCT116 cells in DMEM/10%FBS/PS and incubated for 30 min-4 h as previously published (Eaton et al., 2018). For CPSF73 protein depletion, CPSF73-AID HCT116 cells were incubated with Tetracycline (final concentration 1 μg/mL) in DMEM/10%FBS for 18 h and then IAA was treated for 3 h as previously published (Eaton et al., 2020).

POINT methodology and library prep

The POINT method initially followed the previously described mNET-seq protocol (Nojima et al., 2016) with some alterations. In brief, crude nuclear fraction was prepared from HeLa or HCT116 cells ($1x10^7$ for POINT-seq and POINT-5, $4x10^7$ for POINT-nano) as in the mNET protocol. Then the chromatin pellet was resuspended in NUN1 (20 mM Tris-HCl (pH 7.9), 75 mM NaCl, 0.5 mM EDTA and 50% Glycerol) and treated with modified NUN2 buffer (20 mM HEPES-KOH (pH 7.6), 300 mM NaCl, 0.2 mM EDTA, 7.5 mM $MgCl_2$, 1% NP-40, 1 M Urea, 3% Empigen, 1x protease inhibitor Complete (-EDTA) and 1x PhosSTOP). Note the Empigen-treated chromatin fraction was gently mixed by tube inversion a few times to avoid chromatin aggregation and incubated on ice for 10 min. The chromatin pellet was then isolated by centrifuged at 400 g for 30 s. This was washed with PBS once and then digested in DNase in the following reaction (10 mM Tris-HCl (pH 7.5), 400 mM NaCl, 100 mM $MnCl_2$, 2 U/μL RiboLock and 0.2 U/μL Turbo DNase) at 37°C for 15 min. After DNA digestion, soluble digested chromatin was collected by 13,000 rpm centrifugation for 10 min. The supernatant was diluted tenfold in ice-cold NET-2E buffer (50 mM Tris-HCl (pH 7.4), 150 mM NaCl, 0.05 % NP-40 and 3% Empigen BB) and anti-Pol II antibody-conjugated beads were added. 10 or 40 μg of anti-Pol II antibody (200 or 800 μL of Dynabeads anti-mouse IgG) was used for POINT-seq and POINT-5 or POINT-nano methods. Immunoprecipitation was performed at 4°C for 1 h. The beads were washed with 1 mL of ice-cold NET-2E buffer six times. The isolated nascent RNA was then purified using Trizol reagent technology (Direct-zol) twice with one Turbo DNase treatment at 37°C for 10 min. Size of the RNA was analyzed using 4150 TapeStation (Agilent) with high sensitivity RNA kit according to the protocol. For sequencing the isolated RNA, we employed following methods.

POINT-seq

The isolated RNA was fragmented to 150-200 nt at 90°C for 10 min according to a protocol of NEBNext Ultra II Directional RNA library prep kit to prepare PCR library. Therefore small RNAs (up to 150 nt) were removed during the library prep. The library was applied to Illumina NovaSeq6000 (Novogene UK).

POINT-5

The template switching approach with random N6 primers was applied to the isolated RNA by following the protocol of the SMARTer Stranded RNA-seq kit. The amplified PCR library was size-selected with SPRISelect beads to 150-800 bp and then applied to an Illumina NovaSeq6000 (Novogene UK). Note that no RNA fragmentation is required in this library prep.

POINT-nano

PolyA tails were added to the isolated RNA by *in vitro* polyadenylation with *E.coli* PAP. pA+RNA was then size-selected using SPRISelect reagent (x0.6 volume) to remove RNA smaller than 500 nt. The direct cDNA library prep kit was employed.

Sequencing service

Illumina and ONT sequencing (PromethION) were conducted by the high throughput genomics team of the Wellcome Trust Centre for Human Genetics (WTCHG), Oxford and Novogene UK (Europe Cambridge Branch).

Exoterminator treatment

500 ng of 5′ monophosphate RNA isolated by POINT method was specifically digested with Exoterminator (Cambio) at 30°C for 1 h and then purified with Trizol reagent technology (Direct-zol).

QUANTIFICATION AND STATISTICAL ANALYSIS

Illumina data pre-processing

Quality control for raw short-reads was performed on POINT-seq and POINT-5 data using the FastQC tool. Then, read adaptors were trimmed using TrimGalore in paired-end mode, removing reads with less than 10 nucleotides (nt) and/or low-quality ends (20 Phred score cut-off). The resultant reads were aligned against the reference human genome (GRCh38) using STAR software (Dobin et al., 2013), requiring uniquely mapped reads (–outFilterMultimapNmax 1) and minimum alignment score (–outFilterScoreMin) of 10.

Additionally, for POINT-5 the 5′ end of the original RNA and their directionality was extracted. To do this, the script created for mNET-seq (Nojima et al., 2015) to obtain single nucleotide resolution profiles was adapted to define the 5′ end of the first read in each pair as well as its directionality. Exceptionally, for the POINT-seq DRB experiment, a spike-in SIRV-Set2 RNA was added before library preparation to allow comparison between control and DRB-treated cells. Here, reads were aligned against both SIRV-Set2 sequences available in the Lexogen website (version 170612a) and the reference human genome (GRCh38), using STAR software. Then, reads were counted using SAMtools (Li et al., 2009), considering their directionality based on SAM bitwise flag, and normalized as follows:

$$Normalized\ signal = \frac{HgR \times Sk}{10^6}$$

HgR represents the number of reads aligned against the human genome from a particular region of interest, and Sk represents the total number of read counts aligned against the SIRV-Set 2 sequences. Division by 10^6 was applied to improve readability. To evaluate experimental reproducibility, 2-3 biological replicates were generated. Read counts per replicate for each expressed protein-coding gene were obtained using *BedTools coverage* (Quinlan and Hall, 2010), requiring the same strand for the read and gene (-*s*). Splicing patterns were considered for POINT-seq (-*split*). Furthermore, -*counts* and -*sorted* parameters were added to the command. Spearman's rank-order test was then applied to discover the correlation between samples (ρ). ChrRNA-seq and pA+ RNA-seq were generated as part of GSE60358 and GSE86661; GSE33480, respectively. Their pre-processing was as for POINT-seq. mNET-seq reads were trimmed and aligned as described above for POINT-seq and POINT-5 data. Aligned reads were then transformed into single nucleotide reads by application of script previously created for this purpose (Nojima et al., 2015). Strand-specific CAGE data was pre-processed as described above for POINT-5 data.

POINT-nano pre-processing

Nanopore raw signal fast5 files were base called using Guppy Basecalling 3.0.5 (Oxford Nanopore Technology Ltd.). NanoQC (De Coster et al., 2018) was used for a first evaluation of run sequencing quality. Since several samples were sequenced together, barcodes (NBD104/NBD114) were incorporated into the direct cDNA nanopore reads and identified with qcat. Extracting POINT-nano read directionality is required to determine transcript orientation. Thus, primer GAAGATAGAGCGACAGGCAAGT was searched for in reads using *regEx* Python package, applying the following rules: $i \leq 3$, $d \leq 3$, $s \leq 3$ and $1i+1d+1\ s \leq 4$. Only these reads were preserved with all others discarded, since the Pol II position could not be determined. Barcode and primer sequences in validated reads were trimmed with Porechop, with–*discard_middle* mode on. Subsequently those were aligned with *minimap2* (Li, 2018) with -*ax splice* parameters. *Unmapped reads*, *not primary alignment* or *supplementary reads* were discarded using SAMtools bitwise flag 2308. To prevent contamination from non-authentic 3′ ends due to oligodT priming on internal A rich sequences, reads with any T either in first 2 mapped nt or 3 out of 5 in the same region were ignored for downstream analyses. Finally, reads with 5′ end soft clips longer than 50 nt were discarded. Pol II location was determined by the left most coordinate of the read, extracted with pysam package. Classification of Pol II position over the classes *TSS*, *Exon*, *Intron*, *SS*, *TES* and *Post-TES* was performed using *BedTools Intersect* after extracting these regions in a BED file format for expressed genes. While *TSS* and *TES* classes were classified over a 50 nt region in both directions, SS was defined over a 10 nt region. Post-TES region was determined by [TES+50, TES+550].

Identification of expressed genes

To identify expressed genes in HeLa S3 and HCT116 cells, strand-specific pA+ RNA-seq data from previously published studies (HeLa S3: GSE86661; HCT116: GSE33480) was employed. Adaptors were trimmed with TrimGalore using the same parameters as in POINT-seq pre-processing. Then, *Kallisto* (Bray et al., 2016) mapped the reads against the human transcriptome (Ensembl v90), and TPM measurement for each transcription unit (TU) from the output was acquired. The transcript with highest TPM was selected per gene. Genes having no transcript with TPM higher than 4 were discarded. Moreover, filtered TUs must have protein-coding tag as a biotype, which was extracted from Ensembl GTF file version 90. To better detect signal levels from POINT technology in different experiments, overlapping TUs were excluded. To do this, an extra window of 500 nt upstream and 2000 nt downstream of each TU was added. A final number of 6341 and 5028 genes was identified as expressed in HeLa S3 and HCT116, respectively. Exceptionally metagene side windows of 2 kb upstream and 7 kb downstream were employed to exclude overlapping TUs. This led to 4546 and 5028 genes for HeLa S3 and HCT116, respectively. U snRNAs https://www.genenames.org/cgi-bin/genegroup/download?id=849&type=node, and histone genes https://www.genenames.org/cgi-bin/genegroup/download?id=864&type=branch, are described as part of *HUGO Gene Nomenclature Committee* platform. TUs from these classes without POINT reads were excluded from analyses. tRNAs coordinates were extracted from GtRNAdb 2.0 (Chan and Lowe, 2016), and rRNAs from Ensembl GTF version 90. For analysis, only non-overlapping TUs were considered and were obtained employing bedtools intersect function.

Metagene Analysis

Metagenes were used to represent average Pol II, RNA 5′ ends and RNA distribution levels along genes and their flanking regions. To generate them, pre-processed BAM files, split by forward and reverse strand were used as input for deepTools *bamCoverage* function. A black list of regions for hg38 assembly has been described (https://www.encodeproject.org/files/ENCFF419RSJ/), and all the reads from these were discarded. Also, RPKM normalization was applied. Then, all expressed genes were scaled to

have their TSS and TES overlapping, in a bin size of 10 nt, using the function deepTools *computeMatrix* (Ramírez et al., 2014) in *scale-regions* mode. Signal was captured not only from the gene, but also from 2 and 7 kb upstream and downstream flanking regions, respectively. In a final step, signal was displayed using the deepTools *computeMatrixOperation*, which generated one final table per strand containing the signal for each gene per bin. These tables were the input for a second phase of processing in R script. Here, genes with no signal in all bins and all conditions were removed as well as 1% of genes with the highest and lowest signals, as these could contribute to false average profiles. Finally, ggplot was used to graphically create the metagene.

Heatmaps

Heatmaps were employed to facilitate closer scrutiny of POINT-5 or CAGE-seq 5′end signal for each region individually. Three different heatmap groups were built: mRNA-mRNA, PROMPT-mRNA and eRNA-eRNA, extracted from a previously published study (Chen et al., 2016). As with metagenes, read counts were captured from these datasets in HeLa S3 cells, using deepTools *bamCoverage* function, followed by deepTools *computeMatrix,* but in *reference-point* mode, preserving a bin size of 10 nt. Midpoint was obtained as the equidistant coordinate to the TSSs of both TUs. Regions with no signal in all conditions were removed. The obtained signal from the minus strand was multiplied by −1, and then summed to the signal from the positive strand. This was then scaled using *rescale* function from *scales* R package. Lastly, *geom_raster* from ggplot package was employed to create the heatmap plot.

Cleavage ratio

Cleavage Ratio and Termination index were computed for POINT-5 data in a single-nucleotide basis. Cleavage Ratio was defined as:

$$CR = \frac{TES}{TSS + TES}$$

where TSS is the read counts per kilobase per million reads (FPKM) in the interval [TSS-50, TSS+50] and TES the FPKM for [TES-50, TES+50].

Termination index

The Termination Index was given by:

$$TI = log2\left(\frac{\frac{[TES,\ TES + 2000]_{counts}}{2000}}{\frac{GB_{counts}}{length_{GB}}}\right)$$

where GB stands for genome body and $length_{GB}$ is the number of bp between TSS and TES coordinates.

Premature transcription termination (PTT) analysis

Premature transcription terminated (PTT) genes were identified by comparison between control DMSO and PlaB POINT-seq data, using a simulation basis approach. Each gene was divided into 10 bins. FPKM of each bin for DMSO was measured ($FPKM_{real}$). All reads overlapping the gene under PlaB condition were also randomly sampled, and the FPKM for each bin was measured ($FPKM_{simulation}$). This simulation was repeated 5000 times for each gene. To obtain more robust results and eliminate potential false negative hits, $FPKM_{simulation}$ was divided by a 3.5, value discovered by manual curation, giving rise to $FPKM_{T\_simulation}$. For each simulation, $FPKM_{T\_simulation}$ was compared with $FPKM_{real}$ for each bin and counted for how many times $FPKM_{T\_simulation}$ was lower than $FPKM_{real}$. Starting from the first bin for each gene, whenever $FPKM_{T\_simulation}$ was found lower than $FPKM_{real}$ in at least 90% of the simulations for one bin, the search ended. This led to the conclusion that PTT occurs in that bin region. PTT genes were classified into Early (E), Middle (M), Late (L), according to where the bins were identified. Thus, Early (E) PTT occurred in the first 3 bins, and Middle (M) and Late (L) PTT, occurred between 4-6 bins and 7-9 bins, respectively. If PTT was ascribed to the last bin or was not found for any of the bins then it was considered non-PTT and labeled NC.

Splicing analyses with POINT technology

POINT-nano and POINT-seq data were used to dissect splicing kinetics. Only internal introns were considered for these analyses. Thus, genes with less than 3 introns were discarded, as well as first and last introns from genes with higher intronic complexity. Intron and exon sizes were extracted from Ensembl annotations GTF files for expressed genes previously identified. The 25% shortest and longest features only were taken to perform splicing comparative analyses. Two-sided Mann-Whitney test was deduced to obtain their significance, followed by a p values adjustment using Holm method. Furthermore, SS strength score was measured with *MaxEntScan* (Yeo and Burge, 2004) using default parameters. Despite the different nature of Nanopore and Illumina reads, splicing status for these reads was accomplished similarly, where the nascent RNA continuity over 3’ SS was evaluated. Thus, reads were classified as unspliced for an intronic event if that continuity was observed, or otherwise as spliced. In detail, several filters and transformations were applied to POINT-nano reads to reveal their splicing status per overlapped intron. First, reads must span 10 nt from 3’ SS to the downstream exon. This overlap was validated with *BedTools intersect*, with prior transformation of reads

from BAM format to BED format using *BedTools bamtobed* in split mode (-split). A full overlap (-F 1) and a shared strand between read and exon (-s) was required here. Then, two different windows of 10 nt were created in addition to downstream 3' SS, upstream 3' SS and 5' SS, to analyze splicing patterns. Reads were considered unspliced when detected in the upstream 3′SS window. All reads which were not detected in this window, but only in upstream 5' SS, were considered spliced. Thus, the splicing fraction denotes the number of reads found as spliced divided by spliced and unspliced reads for a given distance or location of Pol II. Importantly, reads spanning several genes were discarded. Only introns with their 3' SS 1500 bp upstream to Pol II were considered, except for Figures 6 and S6 were a distance of 3500 bp was accepted. Additionally, only the latest fully transcribed intron was considered, according to Pol II position. Exceptionally for Figure 6F, this was repeated for the 2 or 3 latest transcribed introns. Splicing status of POINT-seq reads for each intron was obtained as for POINT-nano, but by use of 5bp windows. For introns with more than 10 spliced and/or unspliced reads, the splicing fraction was measured individually for each intron. Distance to TES from 3' SS was extracted from Ensembl human reference annotation, by subtracting its coordinates and assuming that the TES location is the end coordinate of annotated genes.

Alternative splicing events and cassette cases identification

Constitutive and alternative splicing event classifications were obtained from Ensembl annotations, as previously described (Nojima et al., 2018a). For cases of cassette exon identification, previously published pA+ RNA-seq data from HeLa S3 cells (GSE86661) was analyzed using *vast-tools*. Exon skipping events were isolated, and exons were considered included or excluded when the Ψ value was higher than 0.75 or lower than 0.25, respectively.

Signal extraction and data visualization

Read Coverage and gene annotation manipulations were performed with *BedTools*. BAM files were split by strand with SAMtools according their bitwise flags. In POINT-seq data, forward oriented strand had 83 and 163 flags associated while reverse oriented strand had 99 and 147 flags associated. Oppositely, 99 and 147 flags for POINT-5 correspond to the forward strand, while 83 and 163 to the reverse strand. For POINT-nano, 0 and 16 flags were used to call forward and reverse strand reads, respectively. Data was visualized applying *genomeCoverageBed* function of *BedTools* to each strand independently. Trackhubs in the UCSC browser were created by employing the UCSC bedGraphToBigWig tool (Kent et al., 2002).

Reads quantification

Reads were counted for regions of interest with *BedTools Intersect*, using post normalization to library size and gene length using *read counts per kilobase per million reads* (FPKM).

P values and significance tests

Significance between control and treatment condition was obtained using a two-sided Mann-Whitney test, followed by a p values adjustment using the Holm method. For multiple samples one-way ANOVA comparison was tested, followed by a post hoc analysis using Turkey's test.

Molecular Cell, Volume *81*

Supplemental information

POINT technology illuminates the processing of polymerase-associated intact nascent transcripts

Rui Sousa-Luís, Gwendal Dujardin, Inna Zukher, Hiroshi Kimura, Carika Weldon, Maria Carmo-Fonseca, Nick J. Proudfoot, and Takayuki Nojima

SUPPLEMENTARY INFORMATION

POINT TECHNOLOGY ILLUMINATES THE PROCESSING OF POLYMERASE-ASSOCIATED INTACT NASCENT TRANSCRIPTS

Rui Sousa- Luís[1, 6], Gwendal Dujardin[2], Inna Zukher[2], Hiroshi Kimura[3], Carika Weldon[4], Maria Carmo-Fonseca[1,7], Nick J Proudfoot[2,7], and Takayuki Nojima[2,5,6,7,8]

[1]Instituto de Medicina Molecular João Lobo Antunes, Faculdade de Medicina, Universidade de Lisboa, Av. Professor Egas Moniz 1649-028, Lisboa, Portugal

[2]Sir William Dunn School of Pathology, University of Oxford, South Parks Road, Oxford, OX1 3RE, UK

[3]Cell Biology Centre, Tokyo Institute of Technology, 4259 Nagatsuta-cho, Midori-ku, Yokohama, Kanagawa 226-8503, Japan

[4]Wellcome Trust Center for Human Genetics, University of Oxford, Roosevelt Drive, OX3 7BN, UK

[5]Medical institute of Bioregulation, Kyushu University, 3-1-1 Maidashi, Higashi-ku, Fukuoka, 812-8582, Japan

[6]Co-first authors

[7]Co-corresponding authors

[8]Lead contact

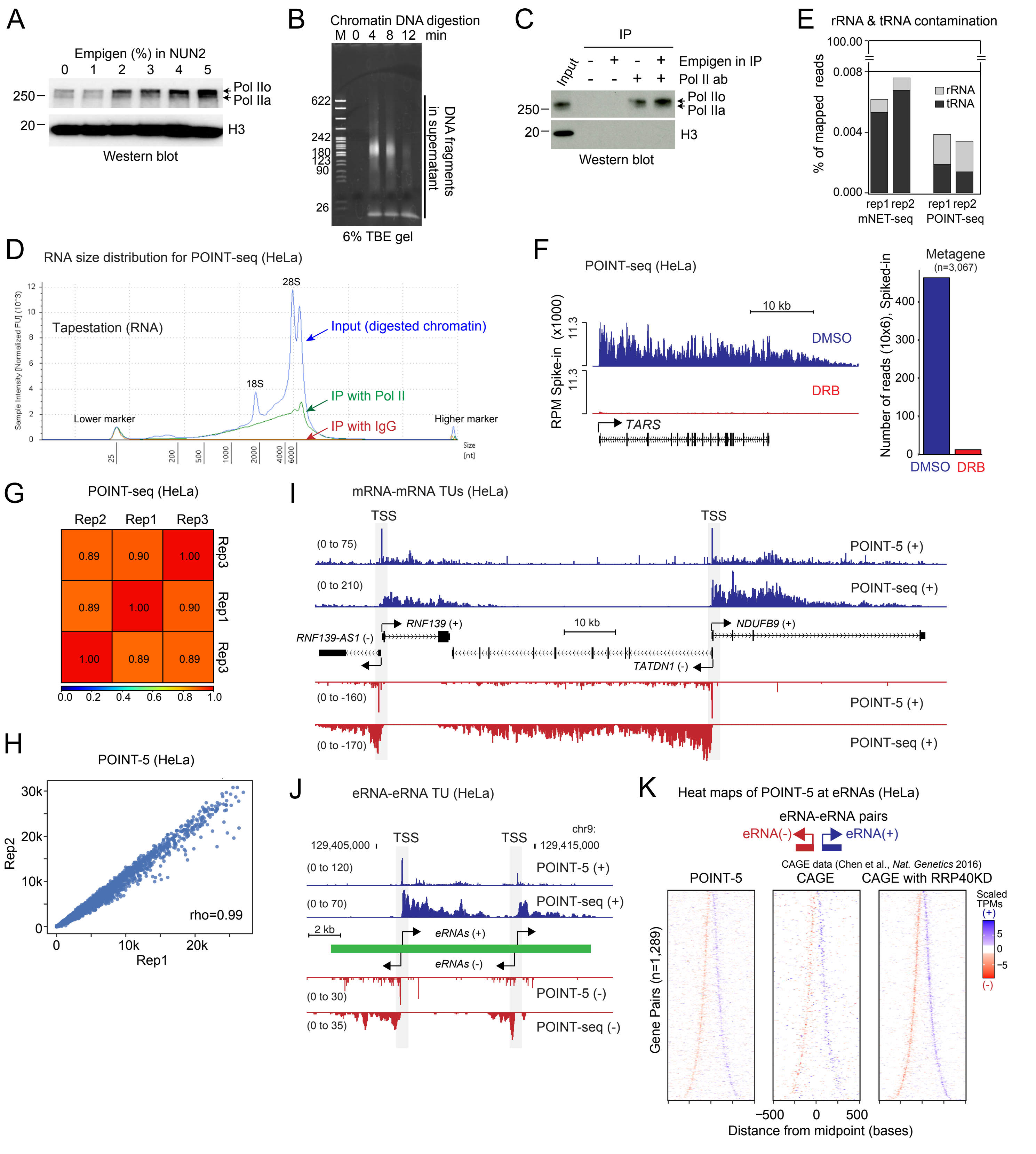

Figure S1. Additional data for POINT-seq and POINT-5 methods (Related to Figure 1)
(A) Western blot of supernatant from DNase-digested HeLa chromatin treated with indicated concentration of Empigen in NUN2 buffer. Antibodies used against Pol II CTD (CMA601) and histone H3. Pol IIo denotes phosphorylated while Pol IIa denotes unphosphorylated isoforms
(B) Gel image of DNA fragments in supernatant of DNase-digested HeLa chromatin treated with 3% Empigen in NUN2 for indicated incubation times.
(C) Western blot of supernatant from DNase-digested HeLa chromatin (input) and IP with anti-Pol II CTD antibody. Antibodies for western blot were anti-Pol II CTD (CMA601) and anti-histone H3.
(D) Tapestation image of POINT RNA size distribution. Digested chromatin fraction as input (blue), IP with mouse IgG (red), and IP with anti-Pol II CTD antibody CMA601 (green).
(E) Stacked barplot showing the percentage of mapped reads overlapping rRNA and tRNA TUs for both mNET-seq and POINT-seq.
(F) (Left) *TARS* of example of POINT-seq signals and (Right) quantification of POINT-seq metagene signals of nonoverlapping PC genes (n=3067) from DMSO or DRB (4 h) -treated HeLa cells.
(G) Heat map for reproducibility between three biological replicates of POINT-seq in HeLa cells.
(H) Scatter plot for reproducibility between two biological replicates of POINT-5 in HeLa cells.
(I) Example view of POINT-5 and POINT-seq signals for mRNA-mRNA pairs in HeLa cells.
(J) Example view of POINT-5 and POINT-seq signals for eRNA-eRNA pairs in HeLa cells.
(K) Heat maps of POINT-5 and CAGE (-/+ RRP40KD) for eRNA-eRNA pairs in HeLa cells. Scaled Transcripts Per Million (TPM) is shown for sense (+, blue) and antisense (-, red).

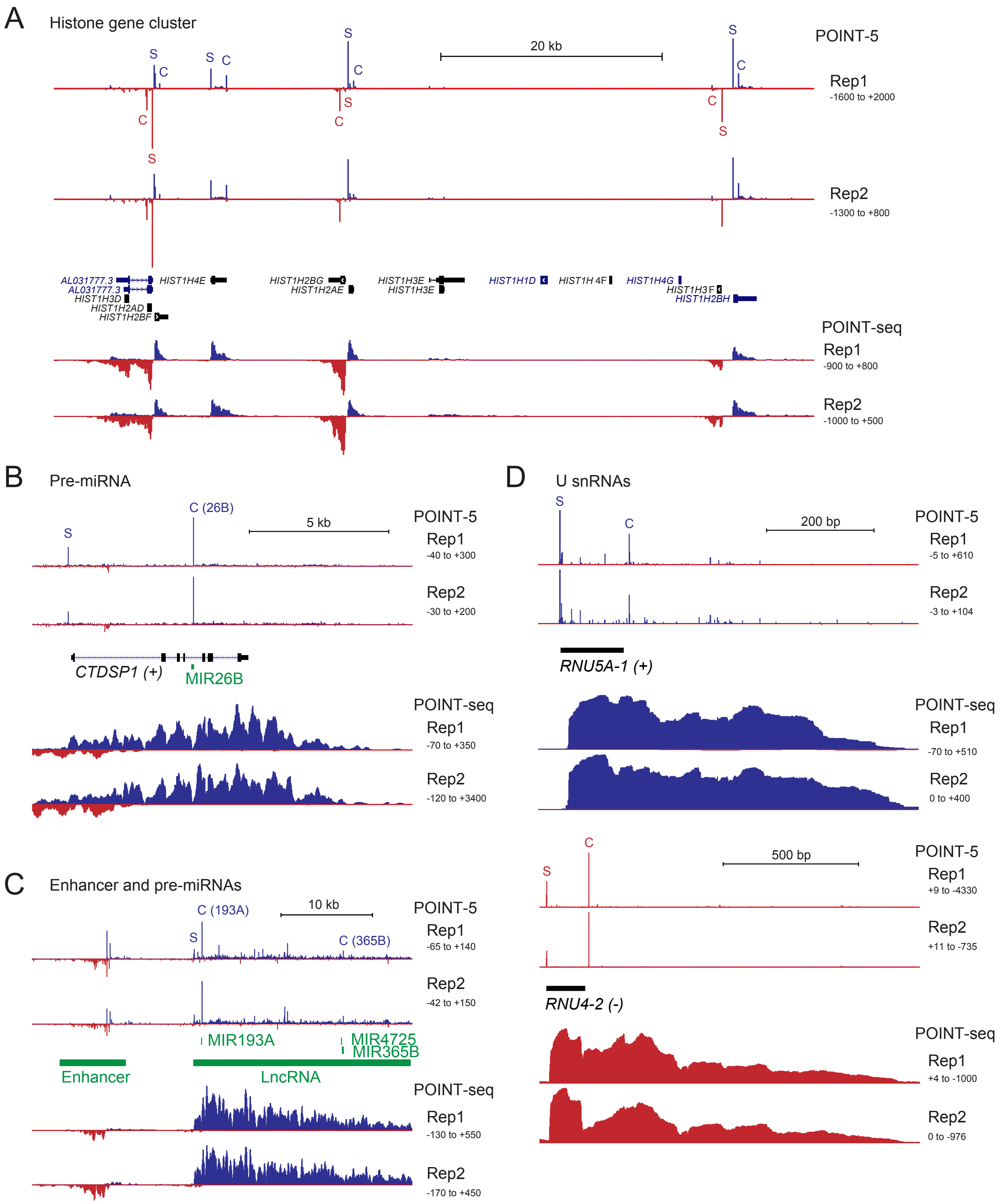

Figure S2. Duplicates of POINT-5 and POINT-seq gene profiles (Related to Figure 2)
(A-D) Example views of POINT-5 and POINT-seq signals on (A) Histone gene cluster, (B) *CTDSP1* as miR26B host gene, (C) Enhancer and unannotated lncRNA with pre-miRNAs, and (D) *RNU5A-1* and *RNU4-2* as U snRNA genes.
Sense strand, blue. Antisense strand, red. S and C are represented as transcription start and RNA cleavage sites, respectively.

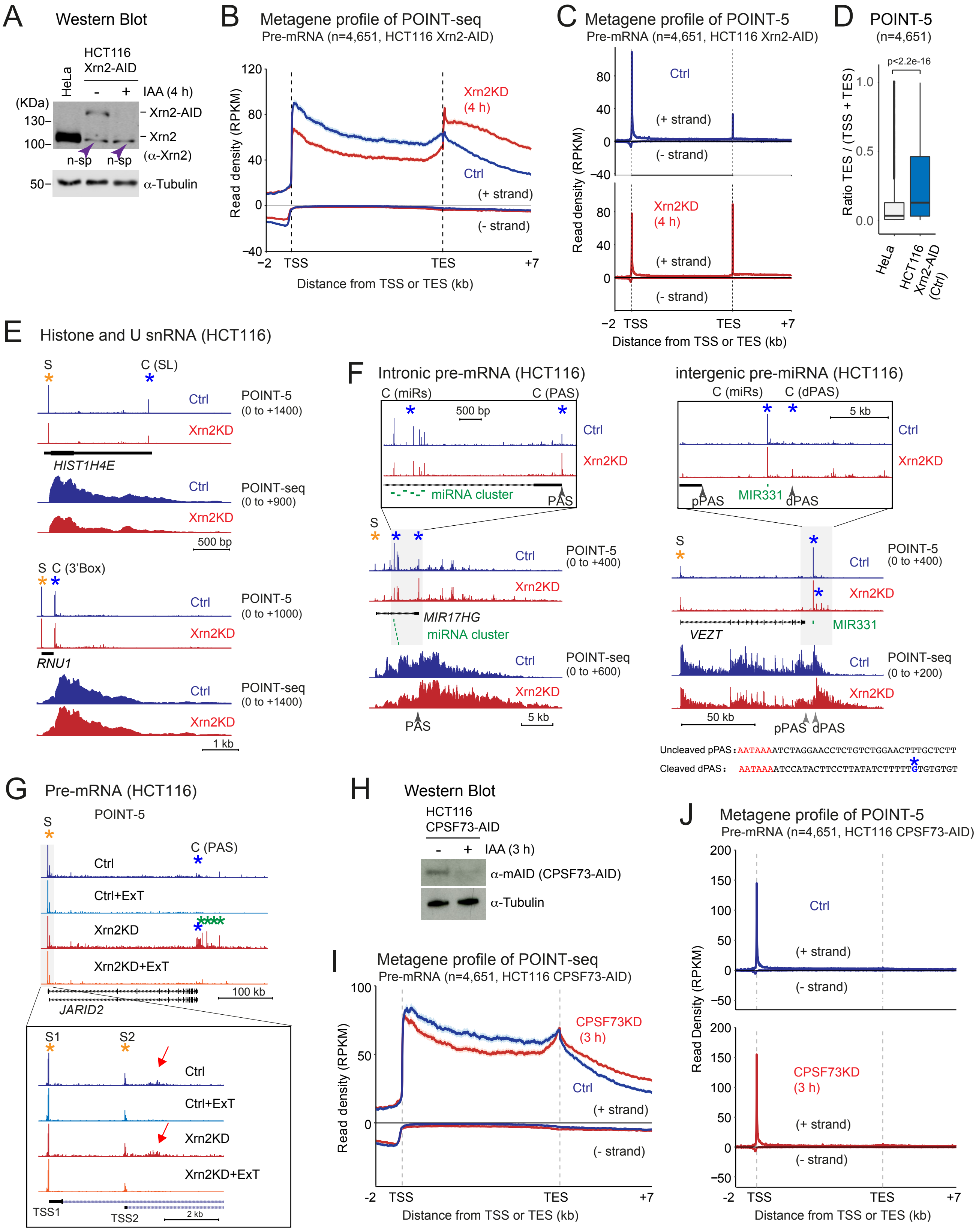

Figure S3. Metagene profile and example genes of POINT-seq and POINT-5 in Xrn2 and CPSF-73 depleted HCT116 cells (Related to Figure 3)
(A) Western blot of whole cell extract from HeLa and HCT116 Xrn2-AID (-/+ IAA, 4 h). Antibodies against Xrn2 and Tubulin. Non-specific (n-sp) bands from anti-Xrn2 antibody indicated by arrow heads.
(B and C) Metagene profile of POINT-seq (B) or POINT-5 (C) on normalised PC genes in HCT116 Xrn2-AID. Ctrl (control) denotes -IAA, KD denotes +IAA for 4 h.
(D) Ratio of TES and TSS on PC genes in HeLa and HCT116 Xrn2-AID Ctrl cells.
(E) *HIST1H4E* and *RNU1* as examples of POINT-5 and POINT-seq on histone and U snRNA genes in HCT116 Xrn2-AID Ctrl and KD cells.
(F) *MIR17HG* and *VEZT* as examples of POINT-5 and POINT-seq on miRNA host genes in HCT116 Xrn2-AID Ctrl and KD cells.
(G) *JARID2* as example of POINT-5 and POINT-seq on PC gene in HCT116 Xrn2-AID Ctrl and KD cells and in vitro ExT treatment. POINT-5 peaks located downstream of PAS in HCT116 Xrn2-AID (KD) cells are indicated as green asterisks. ExT-sensitive POINT-5 peaks located downstream of TSS2 indicated by red arrows.
(H) Western blot of whole cell extract of HeLa and HCT116 CPSF73-AID (-/+ IAA, 3 h). Antibodies against mAID and Tubulin.
(I and J) Metagene profile of (I) POINT-seq or (J) POINT-5 on normalised PC genes (n=4,651) in HCT116 CPSF73-AID Ctrl and KD cells.

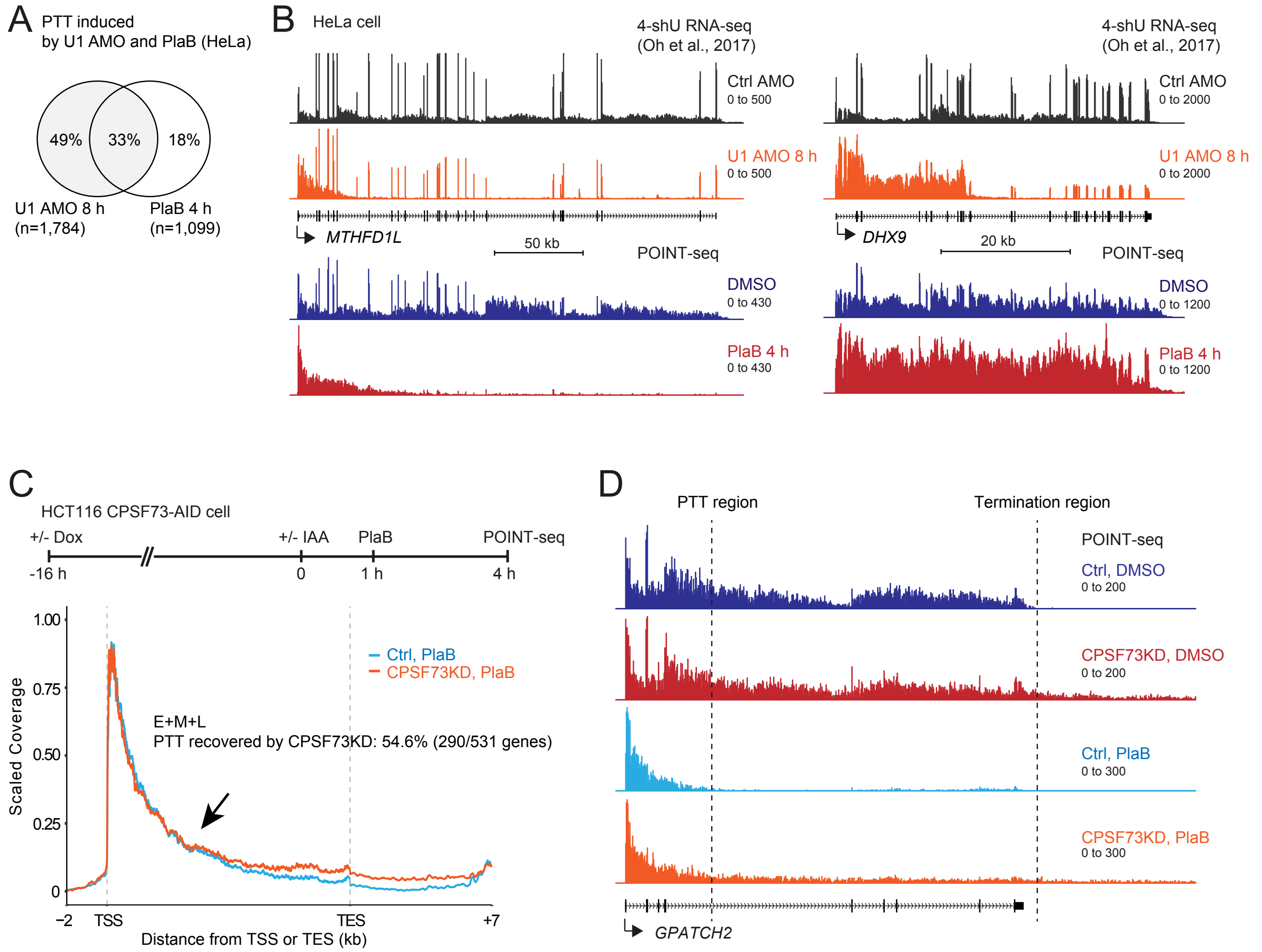

Figure S4. Additional analysis of PlaB induced PTT (Related to Figure 4)
(A) Venn graph of PTT in genes induced by U1 AMO (8 h) or PlaB (4 h) treatment in HeLa cells.
(B) *MTHFD1L* and *DHX9* as examples of 4-shU RNA-seq (Oh et al., 2017) and POINT-seq (this study) signals.
(C) (Top) Schematic of CPSF73KD and PlaB treatment in HCT116 CPSF73-AID cells. (Bottom) Metagene profile of POINT-seq with PlaB (light blue) or CPSF73KD and PlaB (orange) in normalized region from TSS-2kb to TES+7kb. Arrow indicates a starting point of PTT defect induced by CPSF73KD. 290 out of 531 PTT genes were affected.
(D) *GPATCH2* as example of POINT-seq for indicated treatments. The PTT and termination regions are shown as dashed lines.

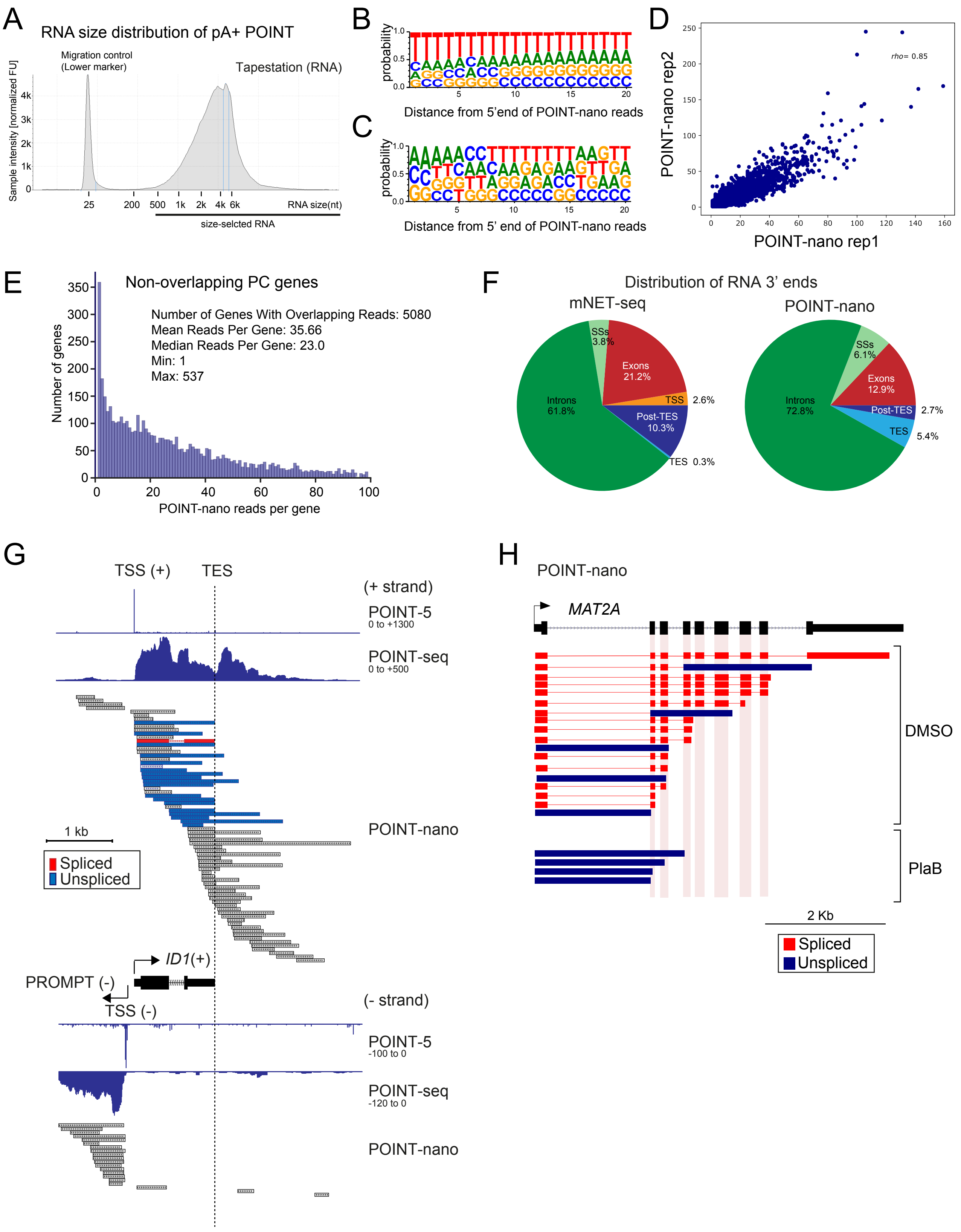

Figure S5. Additional characterization of POINT-nano (Related to Figure 5)
(A) Tapestation image of size distribution of in vitro polyadenylated POINT RNA. The RNA (>500 nt) were size-selected before ONT library prep.
(B and C) Consensus sequences of POINT-nano 5' end reads (B) before or (C) after removal of internal polyA tracts (reverse complement).
(D) Scatter plot for reproducibility between two biological replicates of POINT-nano signals in HeLa cells.
(E) PC gene coverage of POINT-nano reads.
(F) Pie charts of mNET-seq and POINT-nano RNA 3' ends for positions of PC genes.
(G) *ID1* as example of POINT-5, POINT-seq, and POINT-nano signals. Spliced (red), unspliced (blue), other (grey) reads.
(H) POINT-nano reads for *MAT2A* as example of a short PC gene displaying high levels of immediate splicing. Spliced (red), Unspliced (blue).

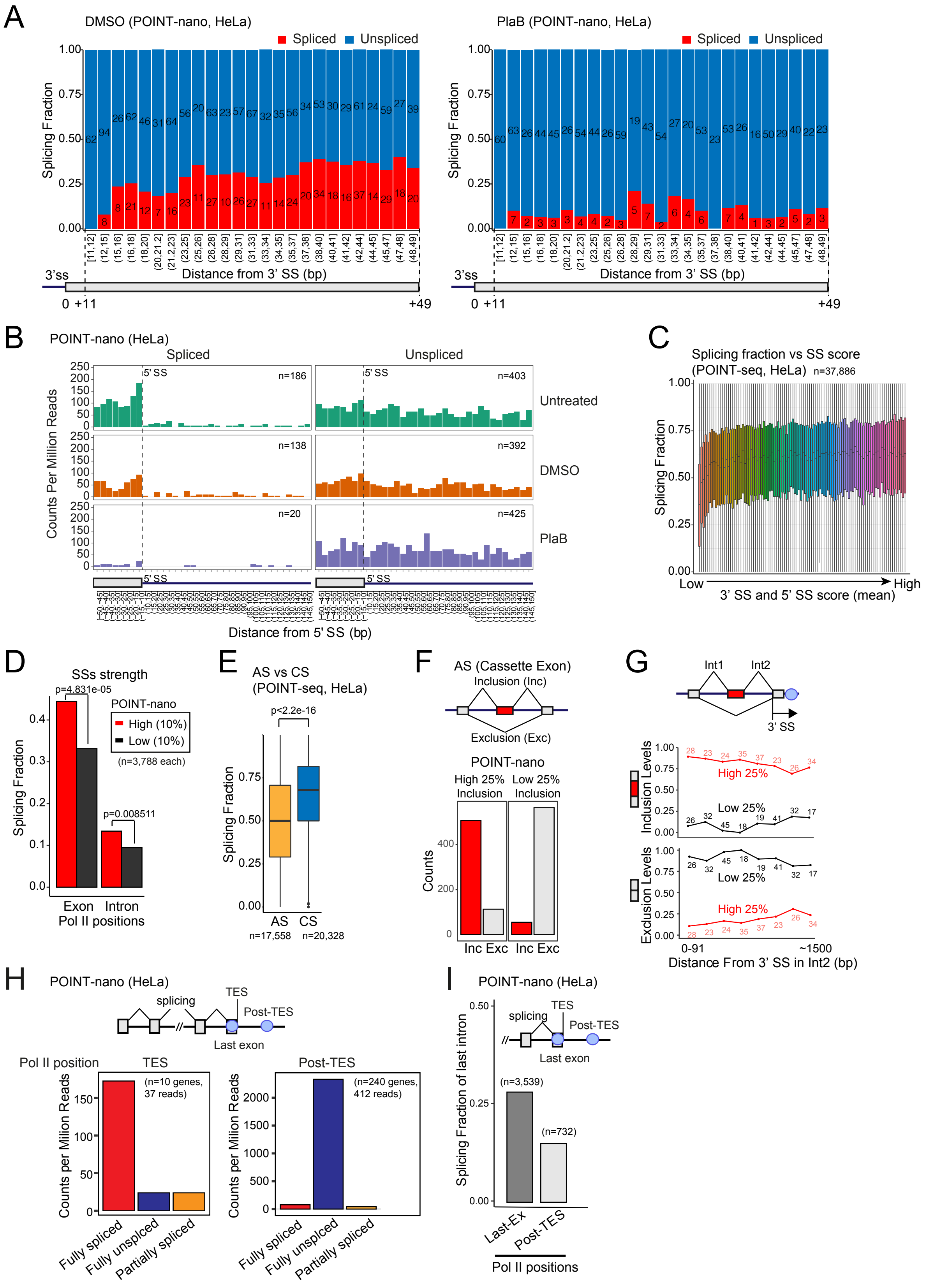

Figure S6. Additional bioinformatics on co-transcriptional splicing kinetics (Related to Figure 6)

(A) Splicing fraction of POINT-nano reads with Pol II located at 3' SS+10 to +49 bp in HeLa cells. Spliced (red), Unspliced (blue).

(B) Counts per million of spliced and unspliced POINT-nano reads when Pol II is located at 5' SS-50 bp to 5' SS+150 bp are shown.

POINT-nano reads from Untreated (Unt, green), DMSO (orange) and PlaB (purple) treated HeLa cells were compared. 5' SSs are indicated by dashed lines. Bin size, 5 bp.

(C) Effect of 3' SS and 5' SS scores (mean) on splicing fraction in POINT-seq analyses.

(D) Effect of 3' SS and 5' SS scores (high 10% or low 10%) on splicing fraction in POINT-nano analyses with Pol II in exon and intron.

(E) Splicing fraction of POINT-seq signals for alternative splicing (AS) or constitutive splicing (CS).

(F) (Top) Cassette exon splicing events classified based on exon inclusion percentage using pA+ RNA-seq data.

(Bottom) Number of exon inclusion (red) and exclusion (grey) events in POINT-nano analyses profiled for high or low 25% exon inclusion event categories.

(G) Exon inclusion or exclusion levels in POINT-nano analyses with Pol II located downstream of 3' SS in intron 2 (Int2). High 25% and low 25% of exon inclusion events are shown as red and black lines, respectively. Numbers of splicing events are indicated.

(H) Counts per million of fully spliced (red), fully unspliced (blue) and partially spliced (orange) POINT-nano reads when Pol II is located at TES or Post-TES region of short genes (<1,500bp) are shown. 10 genes (37 reads) for TES and 240 genes (412 reads) were analysed.

(I) Splicing fraction for last intron removal in POINT-nano analyses. Pol II positions indicated below.

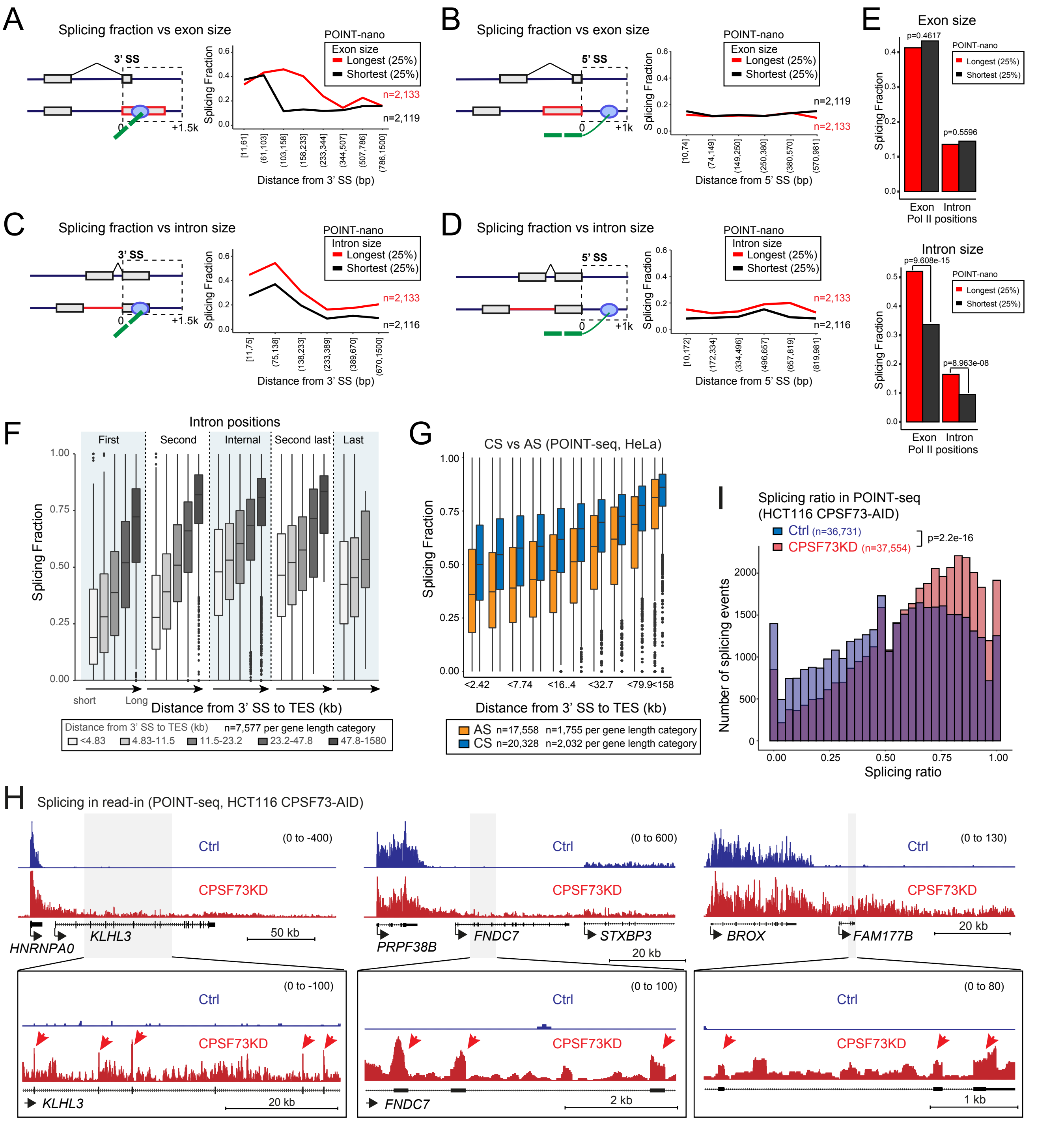

Figure S7. Additional bioinformatics on TU length effect on co-transcriptional splicing (Related to Figure 7)
(A-D) Effect of (A and B) exon or (C and D) intron sizes (long 25%, red or short 25%, black) on splicing fraction in POINT-nano analyses. Pol II is located downstream of (A and C) 3' SS (5' SS+0~1.5 kb) or (B and D) 5' SS (5' SS+0~1 kb).
(E) Quantification of effect of exon (top) or intron (bottom) sizes on splicing fraction POINT-nano signals. Pol II positions are indicated at bottom.
(F) Splicing fraction of POINT-seq signals in HeLa cells. Intron positions and distance from 3' SS to TES (kb) are indicated at top and bottom, respectively.
(G) Splicing fraction of POINT-seq signals in HeLa cells. Distance from 3' SS to TES (kb) is indicated below. AS (yellow), CS (blue).
(H) Examples of read-in transcripts shown by POINT-seq in HCT116 CPSF73-AID cells (-/+ IAA 3 h). Control (blue), CPSF73KD (red). CPSF73KD induced read-in of normally silent *KLHL3*, *FNDC7* and *FAM177B* by active upstream tandem PC genes. Exonic signals derived from splicing are indicated by red arrows.
(I) Number of introns with splicing ratio of POINT-seq signals in HCT116 CPSF73-AID cells (-/+ IAA 3 h). Control (blue), CPSF73KD (light red).
